# C-Terminal Domain of ICA69 Interacts with PICK1 and Acts on Trafficking of PICK1-PKCα Complex and Cerebellar Plasticity

**DOI:** 10.1371/journal.pone.0083862

**Published:** 2013-12-16

**Authors:** Zhen Wang, Ya-Nan Wang, Cheng-Long Sun, Dong Yang, Li-Da Su, Ya-Jun Xie, Lin Zhou, Yin Wang, Ying Shen

**Affiliations:** 1 Department of Neurobiology, Key Laboratory of Medical Neurobiology of the Ministry of Health, Zhejiang Province Key Laboratory of Neurobiology, Zhejiang University School of Medicine, Hangzhou, P. R. China; 2 Department of Neurobiology, Center of Scientific Technology, Cranial Cerebral Disease Laboratory, Ningxia Medical University, Yinchuan, P. R. China; National Institute of Health, United States of America

## Abstract

**Background:**

PICK1 (protein interacting with C-kinase 1) is a PKC (protein kinase C)-binding protein, which is essential for synaptic plasticity. The trafficking of PKCα-PICK1 complex to plasma membrane is critical for the internalization of GluR2 and induction of long-term depression. ICA69 (islet cell autoantigen 69 kDa) is identified as a major binding partner of PICK1. While heteromeric BAR domain complex is suggested to underlie the interaction between PICK1 and ICA69, the role of C-terminal domain of ICA69 (ICAC) in PICK1-ICA69 complex is unknown.

**Methodology/Principal Findings:**

We found that ICAC interacted with PICK1 and regulated the trafficking of PICK1-PKCα complex. ICAC and ΔICAC (containing BAR domain) might function distinctly in the association of ICA69 with PICK1. While ΔICAC domain inclined to form clusters, the distribution of ICAC was diffuse. The trafficking of PICK1 to plasma membrane mediated by activated PKCα was inhibited by ICA69. This action might ascribe to ICAC, because overexpression of ICAC, but not ΔICAC, interrupted PKCα-mediated PICK1 trafficking. Notably, infusion of maltose binding protein (MBP) fusion protein, MBP-ICA69 or MBP-ICAC, in cerebellar Purkinje cells significantly inhibited the induction of long-term depression at parallel fiber- and climbing fiber-Purkinje cell synapses.

**Conclusions:**

Our experiments showed that ICAC is an important domain for the ICA69-PICK1 interaction and plays essential roles in PICK1-mediated neuronal plasticity.

## Introduction

Trafficking of α-amino-3-hydroxy-5-methylisoxazole-4-propionic acid receptor (AMPAR) is a fundamental mechanism for regulating synaptic plasticity and underlies cellular processes involved in learning and memory [1]. PICK1 (protein interacting with C-kinase) is a PDZ and BAR domain-containing protein that emerges as a PKC (protein kinase C)-binding protein [2]. PICK1 also binds to the C-terminal tail of AMPAR subunits GluR2/3 [3,4]. PICK1-GluR2 interaction is involved in the removal of GluR2 from plasma membrane during the induction of long-term depression (LTD) in hippocampus [5-7] and cerebellum [8,9]. PICK1 is also involved in the constitutive trafficking of AMPARs in basal conditions [6] and recycling of internalized AMPARs back to plasma membrane [10-12], which may explain that hippocampal long-term potentiation (LTP) requires PICK1 [13]. During cerebellar synaptic LTD, it is suggestive that PICK1 brings PKCα close to Ser880 at C-terminus of GluR2 to facilitate phosphorylation of Ser880 [14-16]. The targeted PICK1-PKCα complex to synaptic AMPARs leads to the unbinding of ABP/GRIP [17], which is replaced by PICK1 [11].

As a multi-talented protein, PICK1 interacts with various receptors, transporters, and intracellular proteins [18-21]. It is speculated that these interactions may bring about changes in AMPAR trafficking. Interestingly, Cao et al. [22] identified ICA69 (islet cell autoantigen 69 kDa), a BAR-domain-containing protein, as the major binding partner of PICK1 in CNS. Yeast two-hybrid and co-immunoprecipitation (Co-IP) assays showed that ICA69 and PICK1 form a heteromeric BAR domain complex. In neurons, ICA69 colocalizes well with PICK1 in cell bodies and dendrites [22]. Overexpression of ICA69 redistributes PICK1 from synapses to dendrites and reduces synaptic targeting of AMPARs [22]. It is conceivable that heteromeric complexes of ICA69 and PICK1 tether AMPARs in neuronal dendrites, which may influence the induction of AMPAR-mediated synaptic plasticity.

Besides BAR domain, ICA69 also contains a C-terminal domain (ICAC, ICA69 amino acids 257-480), which shows no apparent homology to other known proteins [22]. While heteromeric BAR domain complex is suggested to underlie the interaction between PICK1 and ICA69 [22], the role of ICAC in PICK1-ICA69 complex is unknown. To gain insights of PICK1-ICA69 complex, we tested the interaction between ICAC and PICK1 using Co-IP, immunocytochemistry, and fluorescence resonance energy transfer (FRET) assays. We found that, besides BAR domain, ICAC was also able to strongly interact with PICK1. The expression of ICA69 or ICAC was sufficient to inhibit the trafficking of PICK1-PKCα complex in HEK293T cells. Perfusion of maltose binding protein (MBP) fusion protein, MBP-ICA69 or MBP-ICAC, in Purkinje cells (PCs) blocked the induction of LTD at parallel fiber (PF)- and climbing fiber (CF)-PC synapses.

## Results

### ICAC is an important component of ICA69

**Figure 1:**
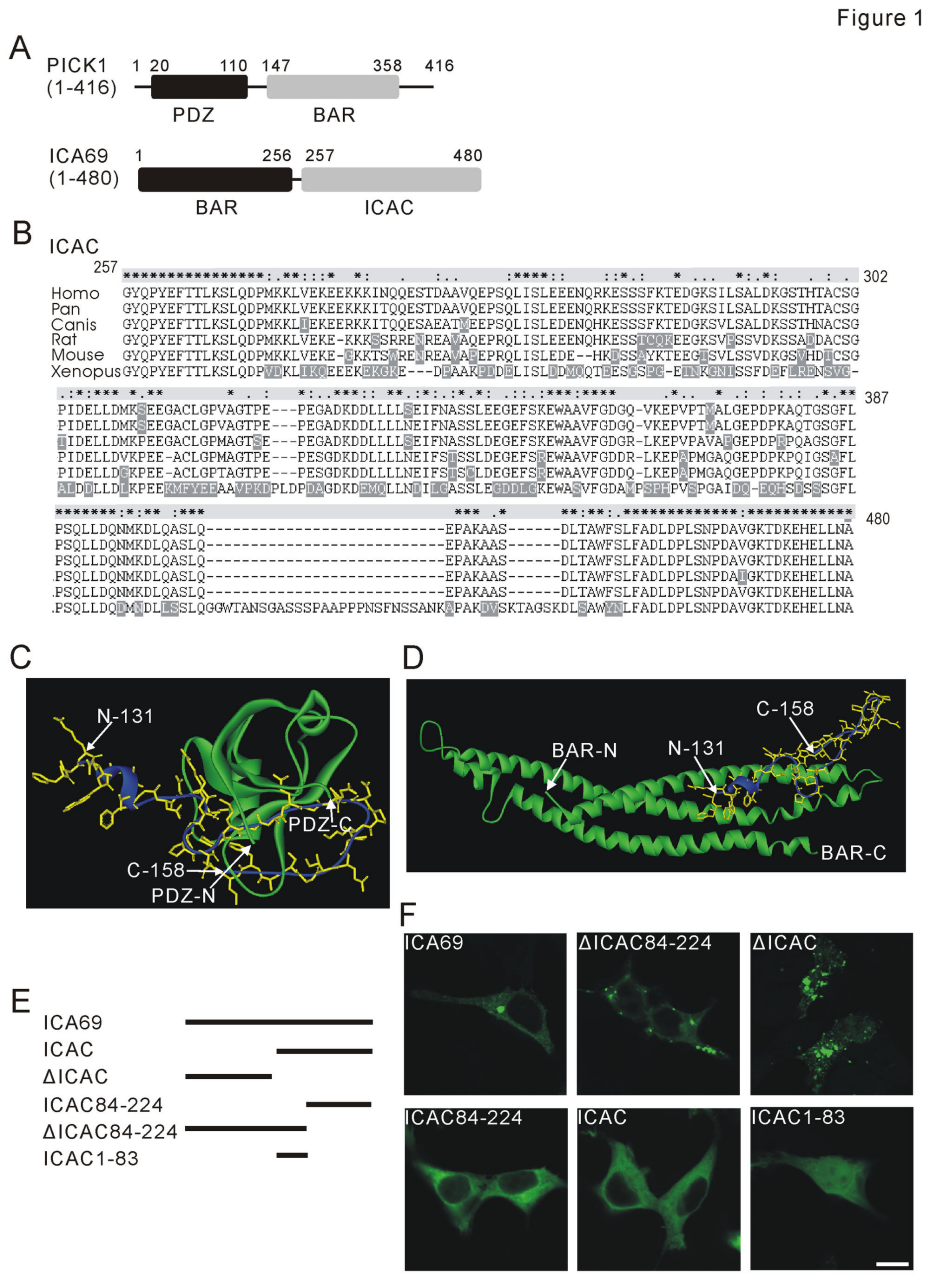
Sequence analysis of ICAC and expression of ICA69 truncations in HEK293T cells. (A) An illustration of domains of rat PICK1 (upper panel) and ICA69 (lower panel). The numbers indicate the locations of amino acids in PICK1 and ICA69. (B) Alignments of ICAC homologues in Homo sapiens (NP-071682; Homo), Pan troglodytes (XP-518971.2; Pan), Canis familiaris (XP-850551.1; Canis), rat (Q63054.2; Rat), Mus musculus (NP-034622.3; Mouse), and Xenopus laevisgi (NP-001084630.1; Xenopus). Above the sequences, stars, colons, and periods indicate that amino acid positions are identical, highly conserved, or weakly conserved. Shadows are distinct residues. (C) A predicted tertiary structural model of interaction between ICAC and PICK1-PDZ (green). ICAC131-158 fragment (yellow: carbon backbone; blue: secondary structure) was predicted to interact with PICK1-PDZ. (D) A predicted tertiary structural model of interaction between ICAC and BAR (green). ICAC 131-158 fragment (yellow: carbon backbone; blue: secondary structure) was predicted to interact with BAR. (E) Schematic diagrams show a series of ICA69 truncation mutants designed, as labeled on left. The numbers indicate the positions of amino acids in ICA69. ΔICAC: 1-256, ICAC: 257-480. (F) Expressions of GFP-tagged ICA69 and its mutants in 293T cells. The upper row shows three different cells expressing ICA69, ΔICAC84-224, or ΔICAC, as labeled. The lower row shows three different cells expressing ICAC1-83, ICAC84-224, or ICAC, as labeled. Scale bar: 10 µm. Data in (F) are representative of more than 4 experiments.

Figure 1A shows main regions of rat PICK1 and ICA69. Previous work showed that BAR domain of ICA69 robustly interacts with PICK1 or BAR domain of PICK1 [22]. Conversely, ICAC alone does not bind to PDZ or BAR domain of PICK1 [22]. Despite lack of evidence, it was also suggested that there might be an interaction between ICAC and PICK1 [22]. This stimulated us to study whether ICAC is involved in the interaction between ICA69 and PICK1. To this end, we used Clustal X software to analyze the sequence of ICAC derived from six representative species (Figure 1B). We found that ICAC was highly conserved across species examined, which implied ICAC may be indispensable for the function of ICA69. Next, structural analysis of ICAC and PICK1 was performed by using ZDOCK and Discovery Studio 2.1 software, based on our previous work [23]. The structure of BAR is quite similar between PICK1 and arfaptin2 [21]. Accordingly, tertiary structure model of PICK1-BAR domain was constructed based on homology modeling implemented in SWISS-MODEL (http://swissmodel.expasy.org/) using the X-ray structure of arfaptin2 (PDB ID: 1I49) as template, as used in previous work [40]. Our predicted models suggested that ICAC can bind to PICK1-PDZ (PDB ID: 2GZV) (Figure 1C) or PICK1-BAR (Figure 1D) at three-dimensional level. We also found that ICAC, spanning 224 amino acids, formed two separate α-helix domains, amino acids (1-83) and amino acids (84-224), which were thereby named as ICAC1-83 and ICAC84-224, respectively. Accordingly, a series of truncations of ICA69 were generated with GFP-tagged at the N-terminus (Figure 1E). GFP fluorescence was observed after the individual plasmid was transfected into 293T cells. As shown in Figure 1F, expression of ΔICAC84-224 or ΔICAC formed numerous clusters in the cytosol while expression of ICA69 only formed seldom clusters. Distinctly, single expression of ICAC, ICAC84-224, or ICAC1-83 was diffuse in cytosol.

### ICAC Interacts with PICK1

**Figure 2:**
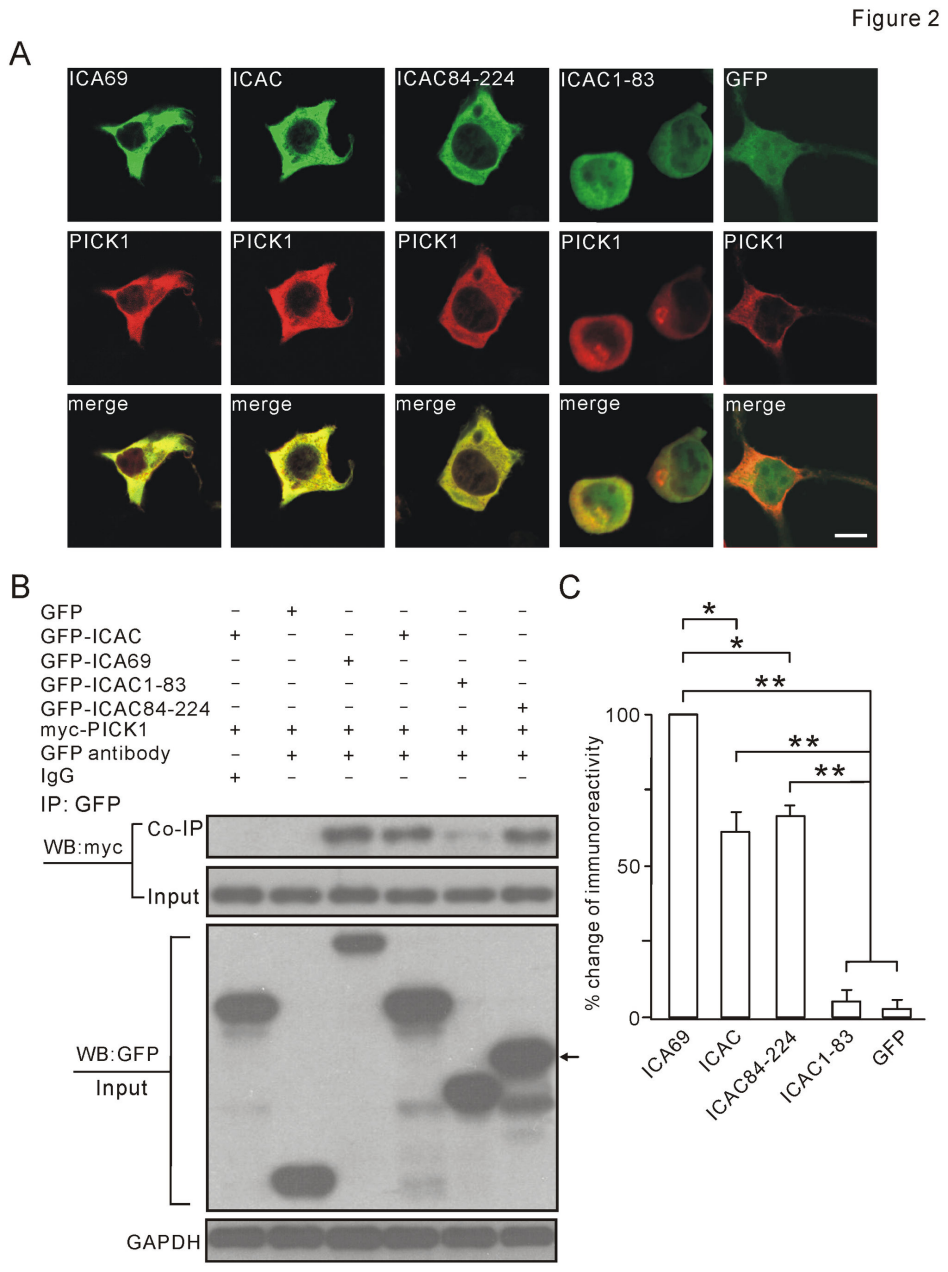
ICAC associates with PICK1 in vitro. (A) 293T cells were co-transfected with myc-PICK1 (red) and GFP-tagged ICA69 or its truncations (green). Note that the expression pattern of PICK1 in cytosol was highly consistent with that of ICA69, ICAC or ICAC84-224. In contrast, expression patterns of ICAC1-83 and PICK1 were quite distinct, as well as GFP. Scale bar: 10 µm. (B) Myc-PICK1 and GFP-tagged ICA69 or its truncations were co-expressed in 293T cells. Constructs transfected in experiments are listed above as “+” or “-”. Cell lysates were immunoprecipitated with anti-GFP antibody. Total expressions of PICK1, ICA69, ICAC, and other truncations are shown in Input lanes as probed by anti-myc or anti-GFP antibody. The arrow on right shows GFP-ICAC84-224 blot. Note that PICK1 was only pulled down by ICA69, ICAC and ICAC84-224, but not ICAC1-83. In negative controls, PICK1 was not pulled down by IgG or GFP. GAPDH was the internal control. (C) Y axis represents the percentage of each precipitated protein normalized to ICA69 group. Percentages were 60±7% (ICAC), 66±5% (ICAC84-224), 11±5% (ICAC1-83), and 6±3% (GFP), which were derived from 4 independent cultures. *P <0.05. **P <0.01.

To examine the interaction between PICK1 and ICAC, GFP, GFP-ICA69, GFP-ICAC, GFP-ICAC1-83, and GFP-ICAC84-224 were individually co-transfected with myc-PICK1 to 293T cells and immunofluorescent staining was performed. PICK1 formed some clusters when it was co-transfected with ICAC1-83 or GFP. Also, the distribution pattern of PICK1 differed from that of ICAC1-83 (Figure 2A). In contrast, PICK1 displayed a diffuse pattern when co-transfected with ICA69, ICAC, or ICAC84-224. Meanwhile, the pattern of PICK1 was very similar to ICA69, ICAC, or ICAC84-224 (Figure 2A). These data suggested that ICAC and ICAC84-224, but not ICAC1-83, may interact with PICK1. 

To confirm the interaction between PICK1 and ICAC, myc-PICK1 was transfected to 293T cells with GFP-ICA69, GFP-ICAC, GFP-ICAC1-83, or GFP-ICAC84-224. Co-IP assay was then performed (Figure 2B). Interaction between PICK1 and truncations was quantified as ratios of Co-IP myc/Input myc, because the efficiency of GFP immunorecipitation was almost same among groups (Figure S1). Each precipitated protein was then normalized to ICA69 group (Figure 2C). Our results indicated that both ICAC and ICAC84-224 had strong binding ability with PICK1, though slightly weaker than ICA69. However, ICAC1-83 had almost no binding ability to PICK1. These results further supported that ICAC and ICAC84-224 interact with PICK1.

### FRET detection of ICAC-PICK1 interaction

**Figure 3:**
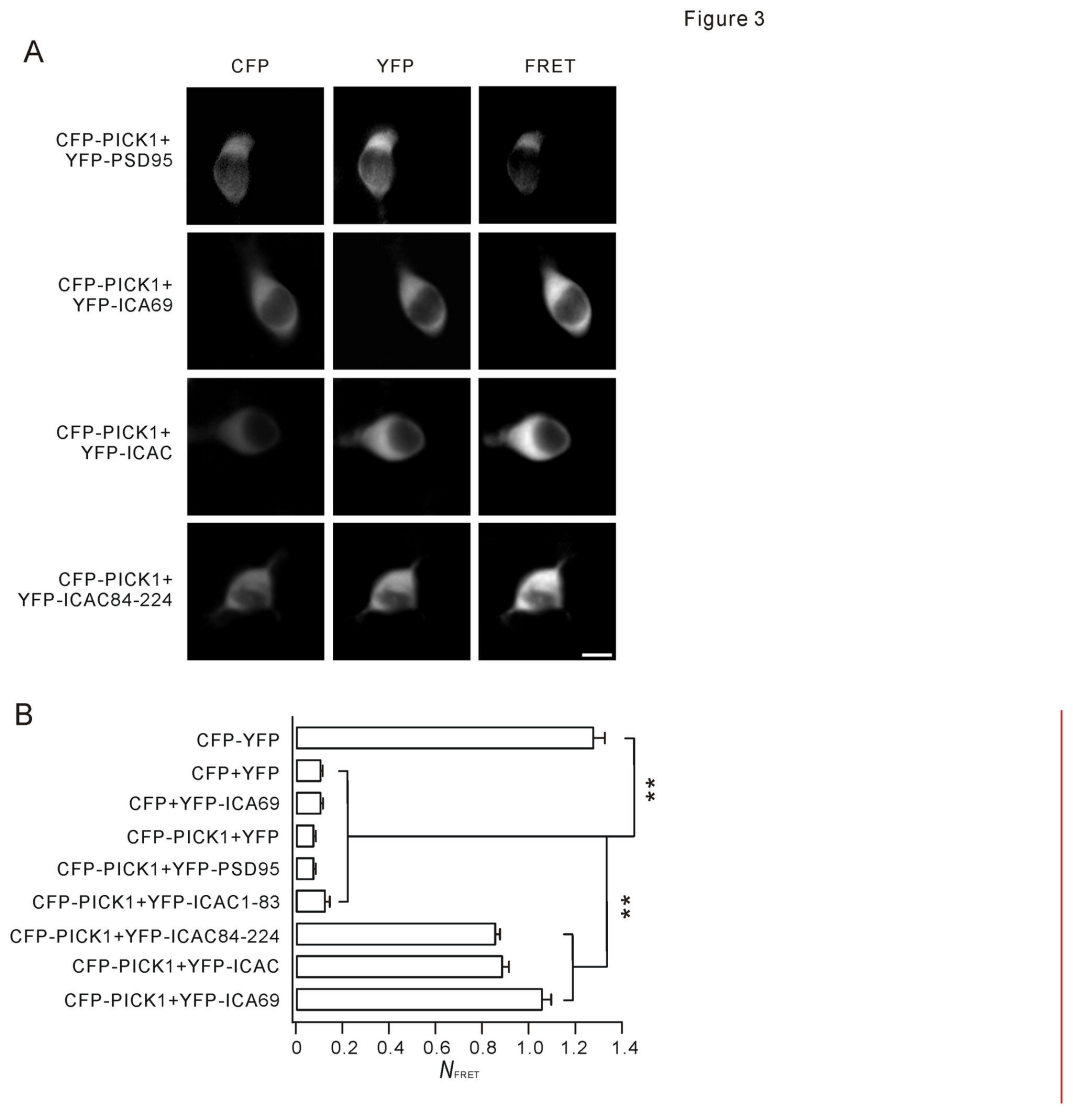
FRET detection of the association of ICAC and PICK1. (A) Fluorescence microscopic images of HEK293T cells expressing fusion proteins indicated on left. Images were made using CFP filter (left column), YFP filter (middle column), or FRET filter (right column). Scale bar: 10 µm. (B) Summary of *N*_FRET_ of different groups. The value of N_FRET_ was proportional to FRET efficiency. When FRET occurs, *N*_FRET_ was greater than 0.2. The values of *N*_FRET_ were 1.28±0.05 (CFP-YFP, n = 66), 0.11±0.01 (CFP+YFP, n = 72), 1.06±0.04 (CFP-PICK1+YFP-ICA69, n = 68), 0.08±0.01 (CFP-PICK1+YFP, n = 54), 0.08±0.01 (CFP-PICK1+YFP-PSD95, n = 57), 0.13±0.02 (CFP-PICK1+YFP-ICAC1-83, n = 56), 0.86±0.02 (CFP-PICK1+YFP-ICAC84-224, n = 68), 0.89±0.03 (CFP-PICK1+YFP-ICAC, n = 66), and 0.11±0.01 (CFP+YFP-PICK1, n = 51). Data were derived from 3 independent experiments. **P <0.01.

FRET has been widely used to measure protein-protein interaction microscopically. Given Co-IP results, we next examined the constitutive association between PICK1 and ICAC in living cells using FRET. Several fluorescent protein-tagged PICK1, ICA69, and ICA69 mutants were constructed, including CFP-PICK1, YFP-ICA69, YFP-ICAC, YFP-ICAC1-83, and YFP-ICAC84-224. Meanwhile, CFP, YFP, YFP-PSD95, and CFP-YFP plasmids were generated as controls. Cells co-expressing CFP-PICK1 and YFP-PSD95 showed no FRET signal (Figure 3A). Distinctly, FRET occurred when CFP-PICK1 was co-expressed with YFP-ICA69, YFP-ICAC, or YFP-ICAC84-224 (Figure 3A). Accordingly, normalized FRET signal (*N*_FRET_) was calculated as described in Eq. 1 for various combinations of co-transfection (Figure 3B). Our results demonstrated that co-expression of CFP-PICK1 with YFP-ICA69, YFP-ICAC, or YFP-ICAC84-224 yielded *N*_FRET_ values significantly larger than negative controls (CFP+YFP, CFP+YFP-ICA69, CFP-PICK1+YFP, and CFP-PICK1+YFP-ICAC1-83). In summary, binding between ICAC and PICK1 was verified by FRET detection. Specifically, ICAC84-224 may be sufficient for ICAC-PICK1 interaction.

### ICA69 does not translocate with activated PKCα

**Figure 4:**
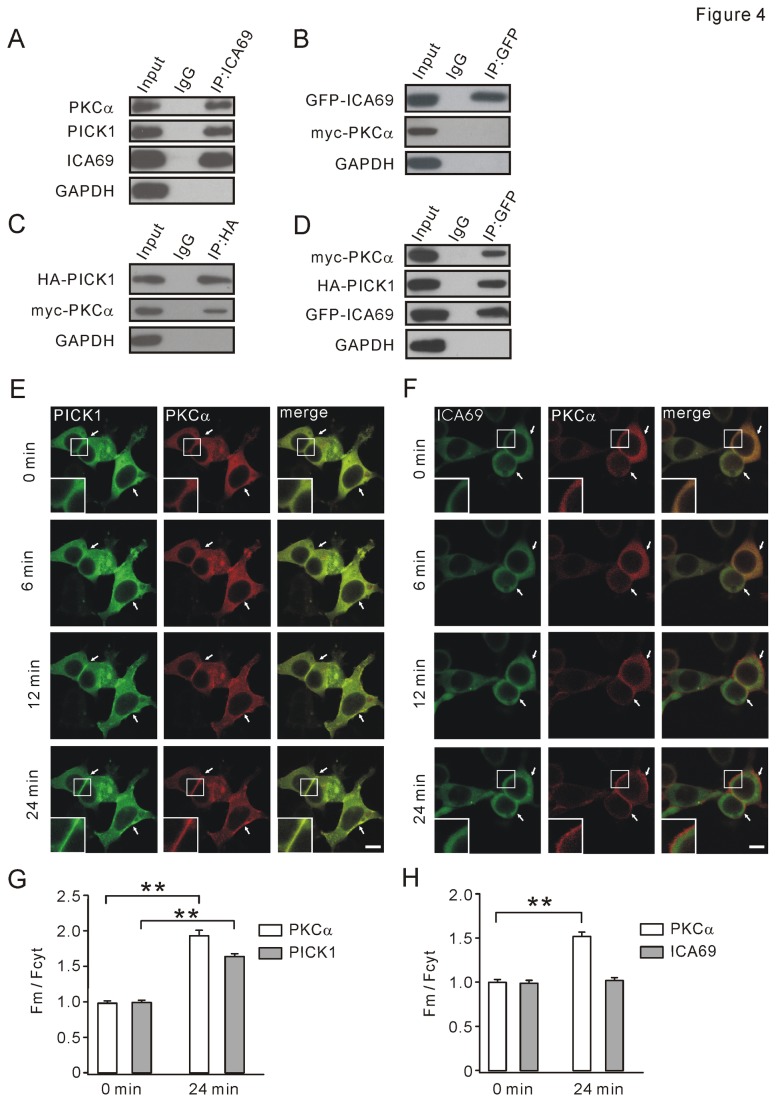
TPA induces the trafficking of GFP-PICK1 to plasma membrane following mCherry-PKCα, with no effect when GFP-ICA69 and mCherry-PKCα were co-expressed. (A) Co-IP was performed with rabbit anti-ICA69 antibody in rat brain homogenate. The antibody pulled down ICA69 itself, and a significant amount of PICK1 and PKCα, indicating that ICA69, PICK1, and PKCα formed complex *in* *vivo*. Rabbit anti- PKCα, mouse anti-PICK1, and rabbit anti-ICA69 antibodies were used for Western blot. Rabbit IgG was the negative control. (B) GFP-ICA69 and myc-PKCα plasmids were co-transfected to 293T cells. GFP-ICA69 was immunoprecipitated using rabbit anti-GFP antibody, indicating that PKCα is not pulled down by ICA69. Rabbit anti-GFP and mouse anti-myc antibodies were used for Western blot. Rabbit IgG was negative control. (C) HA-PICK1 and myc-PKCα plasmids were co-transfected to 293T cells. HA-PICK1 was immunoprecipitated using mouse anti-HA antibody, indicating that PKCα is pulled down by PICK1. Rabbit anti-PICK1 and mouse anti-myc antibodies were used for Western blot. Mouse IgG was negative control. (D) myc-PKCα, HA-PICK1, and GFP-ICA69 were triple-transfected to 293T cells. Both myc-PKCα and HA-PICK1 were immunoprecipitated using rabbit anti-GFP antibody. Mouse anti-PICK1 and anti-myc antibodies were used for Western blot. Mouse IgG was negative control. (E) Activation of mCherry-PKCα by 2 μM TPA induced an increased translocation of GFP-PICK1 and mCherry-PKCα within 24 min to plasma membrane. White arrowheads show typical locations where PICK1 and PKCα were transported to plasma membrane. For images at 0 and 24 min, higher magnifications of plasma membrane (enclosed in small white boxes) were presented to show the translocation of PICK1 and PKCα. (F) GFP-ICA69 did not translocate with mCherry-PKCα when co-expressed in 293T cells after TPA was administrated. Activation of mCherry-PKCα by 2 μM TPA induced a translocation mCherry-PKCα within 24 minutes from cytoplasm to membrane. White arrowheads show the typical locations where PKCα was transported to plasma membrane but ICA69 was retained. For images at 0 and 24 min, higher magnifications of membrane (enclosed in small white boxes) were presented to show the translocation of PKCα but not ICA69. (G) mCherry-PKCα and GFP-PICK1 were co-expressed in 293T cells. F_m_/F_cyt_ values of mCherry-PKCα were 0.97±0.02 (0 min; n = 81) and 1.91±0.06 (24 min; n = 78). F_m_/F_cyt_ values of GFP-PICK1 were 0.98±0.01 (0 min; n = 81) and 1.68±0.04 (24 min; n = 78). (H) mCherry-PKCα and GFP-ICA69 were co-expressed in 293T cells. F_m_/F_cyt_ values of mCherry-PKCα were 1.01±0.01 (0 min; n = 89) and 1.50±0.03 (24 min; n = 80). F_m_/F_cyt_ values of GFP-ICA69 were 1.00±0.01 (0 min; n = 89) and 1.03±0.02 (24 min; n = 80). Scale bars: 10 µm. Data were derived from at least 4 independent cultures. **P <0.01.

The translocation of PICK1-PKC complex to post-synaptic membrane is a critical step for LTD induction [21,24]. Based on our results, we speculated that ICA69 might influence the trafficking of PICK1-PKC complex through binding to PICK1. First, we tested whether ICA69 and PKCα interact with each other. As shown in Figure 4A, anti-ICA69 antibody pulled down both PICK1 and PKCα derived from rat brain homogenate, suggesting that ICA69 may associate with PKCα *in vivo*. Since three-fourths of ICA69 and PICK1 associate with each other in the brain [22], we used *in vitro* Co-IP to determine if ICA69 interacts with PKCα. In this case, GFP-ICA69 and myc-PKCα were co-transfected into 293T cells. IP products indicated that myc-PKCα was not pulled down by GFP-ICA69 (Figure 4B), indicating ICA69 does not bind to PKCα. Additionally, we confirmed the recognized interaction between PICK1 and PKCα [21,24]. HA-PICK1 and myc-PKCα were co-transfected into 293T cells and Co-IP was performed using anti-HA antibody. IP products indicated that myc-PKCα was pulled down by HA-PICK1 (Figure 4C). Finally, triple transfection of myc-PKCα, HA-PICK1, and GFP-ICA69 was performed in 293T cells (Figure 4D). We found that ICA69 was able to interact with PKCα in presence of PICK1. Collectively, these data suggested that ICA69 can interact with PICK1-PKCα complex through PICK1.

PKCα undergoes a rapid time-dependent translocation to plasma membrane in response to TPA (agonist of PKC) stimulation [25]. Activated PKCα binds to PICK1 and transports it to plasma membrane in 293 cells [26]. We utilized this model to study the role of ICA69 in the trafficking of PICK1-PKCα complex. When co-expressed in 293T cells, mCherry-PKCα and GFP-PICK1 appeared homogeneously distributed across cytosol (Figure 4E, top row), consistent with previous work [22,26]. Upon stimulation with 2 μM TPA, GFP-PICK1 translocated with mCherry-PKCα from cytosol to membrane within 6 min (Figure 4E, second row). This phenomenon was more prominent when recording time reached 24 min after TPA stimulation (Figure 4E, third and fourth rows; also see Movie S1). When mCherry-PKCα and GFP-ICA69 were co-expressed in 293T cells, ICA69 formed a few clusters (Figure 4F), similar to single transfection of ICA69 (Figure 1F). Different from PICK1, ICA69 did not follow PKCα to membrane after the treatment with 2 μM TPA. As control, TPA failed to induce a translocation of GFP-ICA69 or mCherry-PICK1 to membrane in absence of mCherry-PKCα (Figure S2). Quantitative analysis was conducted by measuring fluorescence intensity ratio of membrane/cytosol (F_m_/F_cyt_) at 0 and 24 min of time-lapse imaging [26]. When mCherry-PKCα and GFP-PICK1 were co-expressed, F_m_/F_cyt_ values of PKCα and PICK1 were both significantly increased at 24 min (n = 81; Figure 4G). However, F_m_/F_cyt_ value of ICA69 was not altered at 24 min when it was co-expressed with PKCα (n = 80; Figure 4H). These data indicated that TPA-stimulated PKCα can bring PICK1, but not ICA69, to membrane.

### ICA69 or ICAC retains PICK1 but not PKCα in the cytosol facing TPA stimulation

**Figure 5:**
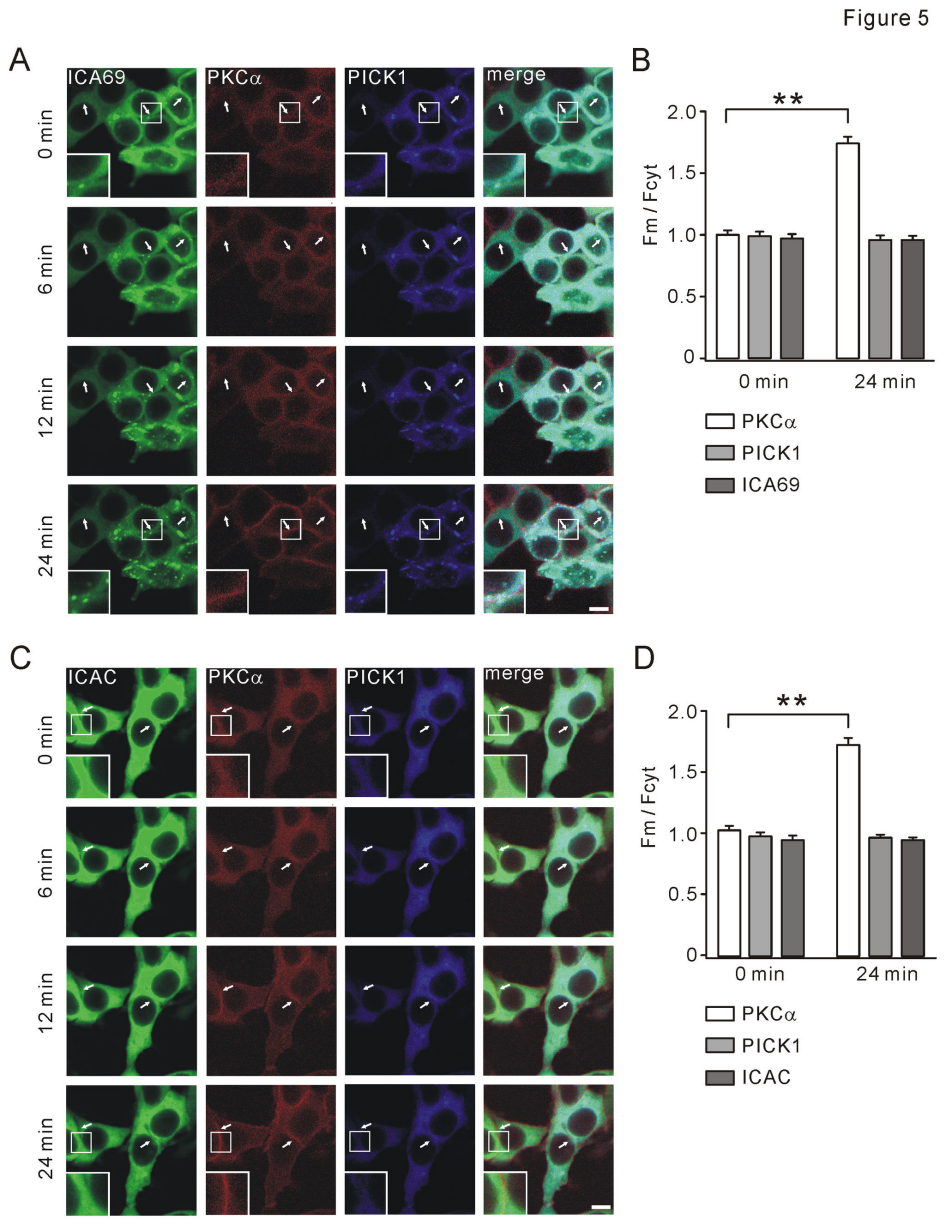
Both ICA69 and ICAC abolish TPA-induced translocation of PICK1. (A) GFP-ICA69, mCherry-PKCα, and CFP-PICK1 were co-expressed in 293T cells. Co-transfection of ICA69 abolished 2 μM TPA-induced translocation of PICK1 but not PKCα. Note that cytosolic clusters of ICA69 and PICK1 were increased in some cells as time elapsed. White arrowheads show typical locations where PKCα was transported to plasma membrane, while ICA69 and PICK1 were retained. For images at 0 and 24 min, higher magnifications of plasma membrane (enclosed in small white boxes) were presented to show the translocation of PKCα, but not ICA69 or PICK1. (B) At 0 min, F_m_/F_cyt_ values of GFP-ICA69, mCherry-PKCα, and CFP-PICK1 were 0.97±0.02, 1.00±0.02, and 0.99±0.02, respectively (n = 84). At 24 min, F_m_/F_cyt_ values of GFP-ICA69, mCherry-PKCα, and CFP-PICK1 were 0.96±0.02, 1.74±0.04, and 0.96±0.02, respectively (n = 82). (C) GFP-ICAC, mCherry-PKCα, and CFP-PICK1 were co-transfected to 293T cells. Co-transfection of ICAC abolished 2 μM TPA-induced translocation of PICK1 but not PKCα. White arrowheads show typical locations where PKCα was transported, while ICAC and PICK1 were retained. For images at 0 and 24 min, higher magnifications of plasma membrane (enclosed in small white boxes) were presented to show the translocation of PKCα but not ICAC or PICK1. (D) At 0 min, F_m_/F_cyt_ values of GFP-ICAC, mCherry-PKCα, and CFP-PICK1 were 0.95±0.02, 1.03±0.02, and 0.98±0.02, respectively (n = 89). At 24 min, F_m_/F_cyt_ values of GFP-ICAC, mCherry-PKCα, and CFP-PICK1 were 0.95±0.01, 1.73±0.04, and 0.97±0.01, respectively (n = 89). Scale bar: 10 µm. Data were derived from at least 4 independent cultures. **P <0.01.

We next investigated whether ICA69 affects the trafficking of PICK1-PKCα complex in response to TPA stimulation. GFP-ICA69, mCherry-PKCα, and CFP-PICK1 were co-expressed in 293T cells. Treatment with 2 μM TPA induced a prominent PKCα translocation from cytosol to membrane within 24 min (Figure 5A, second column). However, neither ICA69 nor PICK1 was trafficked to membrane (Figure 5A, left and third columns; also see Movie S2). As summarized in Figure 5B, F_m_/F_cyt_ value of PKCα was significantly increased at 24 min, but F_m_/F_cyt_ values of PICK1 and ICA69 were not altered (n = 82), suggesting that ICA69 blocks TPA-induced PICK1 translocation. Interestingly, both ICA69 and PICK1 displayed increased cytosolic clusters as time elapsed in some cells (Figure 5A, first and third columns). 

We next examined if ICAC affects the trafficking of PICK1-PKCα complex facing TPA stimulation. Similarly, GFP-ICAC, mCherry-PKCα, and CFP-PICK1 were co-expressed in 293T cells. Treatment with 2 μM TPA induced a PKCα translocation from cytosol to membrane within 24 min (Figure 5C, second column). However, neither ICAC nor PICK1 was trafficked to membrane (Figure 5C, first and third columns). Statistics of F_m_/F_cyt_ values of PKCα, PICK1, and ICAC are shown in Figure 5D (n = 82). Specifically, we did not find any increased cluster of ICAC or PICK1 as time elapsed (Figure 5C, first and third columns). 

### ICAC84-224 retains PICK1 in cytosol upon TPA stimulation

**Figure 6:**
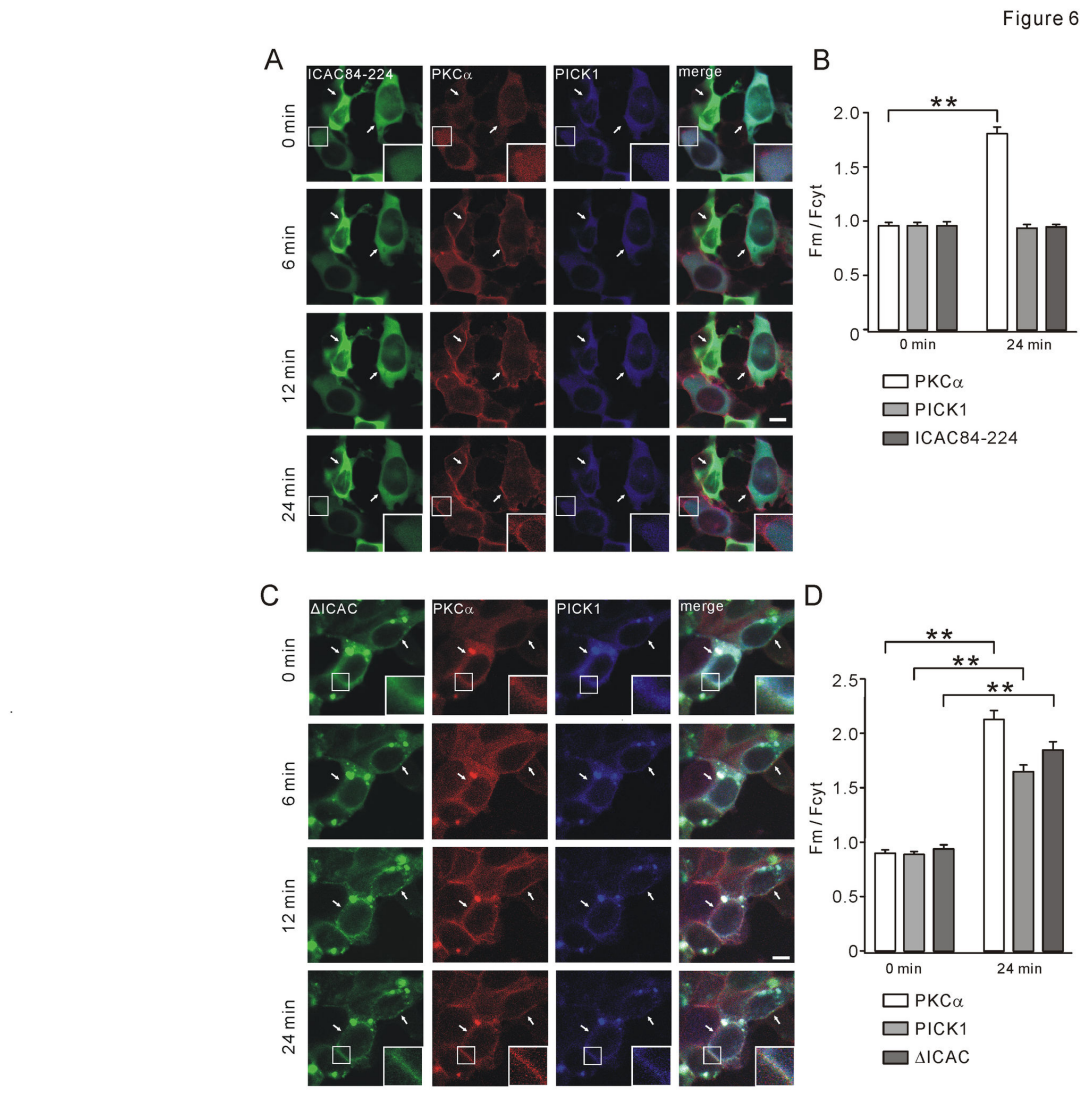
Different roles of ICAC84-224 and ΔICAC in TPA-induced PICK1 translocation. (A) GFP-ICAC84-224, mCherry-PKCα, and CFP-PICK1 were co-expressed in 293T cells. 2 μM TPA induced the translocation of PKCα to the membrane from 6 min to 24 min. Both ICAC84-224 and PICK1 were retained in cytosol and they displayed similar diffuse distribution. For images at 0 and 24 min, higher magnifications of plasma membrane (enclosed in small white boxes) were presented to show the translocation of PKCα but not ICAC84-224 or PICK1. (B) At 0 min, F_m_/F_cyt_ values of GFP-ICAC84-224, mCherry-PKCα, and CFP-PICK1 were 0.96±0.03, 0.96±0.02, and 0.96±0.02, respectively (n = 88). At 24 min, F_m_/F_cyt_ values of GFP-ICAC84-224, mCherry-PKCα, and CFP-PICK1 were 0.95±0.01, 1.81±0.05, and 0.94±0.02, respectively (n = 80). (C) GFP-ΔICAC, mCherry-PKCα, and CFP-PICK1 were co-expressed in 293T cells. 2 μM TPA induced a translocation of them all to the membrane as time elapsed. For images at 0 and 24 min, higher magnifications of plasma membrane (enclosed in small white boxes) were presented to show the translocation of ΔICAC, PKCα, and PICK1. (D) At 0 min, F_m_/F_cyt_ values of GFP-ΔICAC, mCherry-PKCα, and CFP-PICK1 were 0.94±0.03, 0.90±0.02, and 0.89±0.01, respectively (n = 89). At 24 min, F_m_/F_cyt_ values of GFP-ΔICAC, mCherry-PKCα, and CFP-PICK1 were 1.85±0.06, 2.13±0.07, and 1.65±0.05, respectively (n = 80). Scale bars: 10 µm. Data were derived from at least 4 independent cultures. **P <0.01.

ICAC84-224 was shown to possess a tight interaction with PICK1 (Figures 2 and 3). It was interesting to examine whether ICAC84-224 affects the trafficking of PICK1-PKCα complex. GFP-ICAC84-224, mCherry-PKCα, and CFP-PICK1 were co-transfected to 293T cells. TPA treatment (2 μM) induced PKCα translocation to plasma membrane within 24 min (Figure 6A, second column), but neither ICAC84-224 nor PICK1 was trafficked to membrane (Figure 6A, first and third columns; also see Movie S3). Statistics of F_m_/F_cyt_ values of PKCα, PICK1, and ICAC84-224 are shown in Figure 6B (n = 80). These results suggested that ICAC84-224 is sufficient to retain PICK1 in cytosol.

As comparison, effects of ΔICAC and ICAC1-83 on the trafficking of PICK1-PKCα complex were investigated. First, GFP-ΔICAC, mCherry-PKCα, and CFP-PICK1 were co-transfected to 293T cells. Both ΔICAC and PICK1 formed some clusters (Figure 6B, first and third columns), which were also seen when PKCα was not co-transfected (Figure S3). TPA (2 μM) induced obvious translocation of ΔICAC, PKCα, and PICK1 to plasma membrane (Figure 6C), as demonstrated by increased F_m_/F_cyt_ values of ΔICAC, PICK1, and PKCα at 24 min (Figure 6D). Second, GFP-ICAC1-83, mCherry-PKCα, and CFP-PICK1 were co-transfected to 293T cells. Similar to ΔICAC, ICAC1-83 did not retain PKCα and PICK1 in cytosol after TPA (2 μM) stimulation (Figure S4). Distinctly, ICAC1-83 displayed a diffuse distribution all the time during TPA stimulation (Figure S4A, first column), showing, yet again, that ICAC1-83 does not bind to PICK1.

### Infusion of MBP-ICA69/MBP-ICAC in PCs inhibits PF-PC LTD

**Figure 7:**
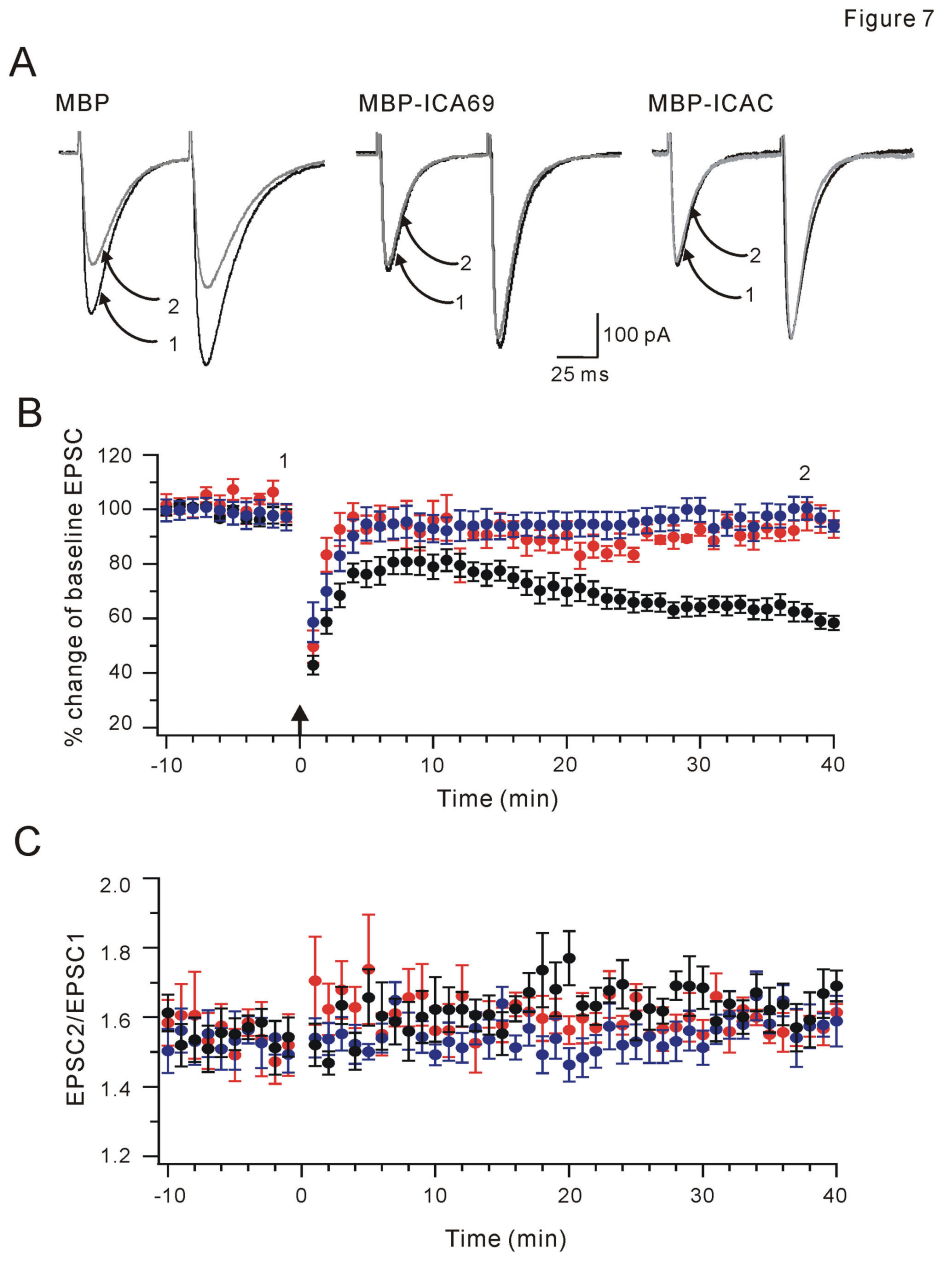
ICA69 and ICAC inhibit PF-LTD. (A) Example traces before and after PF-LTD. Currents indicated by 1 (black) or 2 (grey) were collected at time points indicated in (B). (B) Comparison of three groups of neurons recorded. Mean peak amplitudes of PF-evoked EPSC1 are displayed versus time (Black: MBP, n = 10; Red: MBP-ICA69, n = 10; Blue: MBP-ICAC, n=9). Following a 10-min baseline recording, LTD was induced at t = 0 min. Tetanic stimulation is indicated by the upward arrow. (C) Time courses of PPF of EPSCs. Black: MBP, n = 10; Red: MBP-ICA69, n = 10; Blue: MBP-ICAC, n=9.

How might ICA69 affect cerebellar synaptic plasticity that requires the trafficking of PICK1? To address this question, MBP fusion proteins were purified and added to patch pipettes during whole-cell recordings in cerebellar PCs [8]. Purified MBP backbone, MBP-ICA69, MBP-ICAC, and MBP-ΔICA69 proteins were identified by Coomassie Brilliant Blue staining and Western blots (Figure S5). To ensure that fusion proteins had adequate time to diffuse into cells in whole-cell configuration, experiments begun after a minimum of 15 min following patch ruptures [8]. Series and input resistances were monitored throughout experiments. No obvious change of both resistances were found after ruptures (data not shown), indicating that infusion of fusion proteins does not affect cell condition. Following a stable 10-min baseline recording period, a paired-stimuli protocol including a 5-pulse train stimuli of PFs at 100 Hz and a 100-ms depolarization of PC to 0 mV was administrated. This protocol was repeated 30 times at an inter-burst interval of 2 s [27,28]. When MBP (50 μg/ml) was infused into PCs, paired stimuli induced robust LTD (62±3% of baseline at t = 38 min, n = 10; P <0.01; Figure 7A and 7B). In contrast, perfusion of MBP-ICA69 (50 μg/ml) resulted in a significant attenuation of subsequently-induced LTD (97±5% of baseline at t = 38 min, n = 10; P >0.05; Figure 7A and 7B). Perfusion of both MBP and MBP-ICA69 did not alter paired-pulse facilitation (PPF; Figure 7C). MBP-ICAC fusion protein (50 μg/ml) was also infused into PCs. Similar to MBP-ICA69, MBP-ICAC blocked LTD induction (100±4% of baseline at t = 38 min, n = 10; P >0.05; Figure 7A and 7B) while PPF was not changed (Figure 7C). The function of MBP-ΔICAC in cerebellar LTD was also examined. Purified MBP-ΔICAC was supplemented into pipette solution during whole-cell recordings. Our results showed that MBP-ΔICAC did not affect LTD induction (Figure S6), in consistent with the result that ΔICAC did not retain PICK1 (Figure 6B).

### CF-PC LTD is absent with MBP-ICA69/MBP-ICAC in PCs

**Figure 8:**
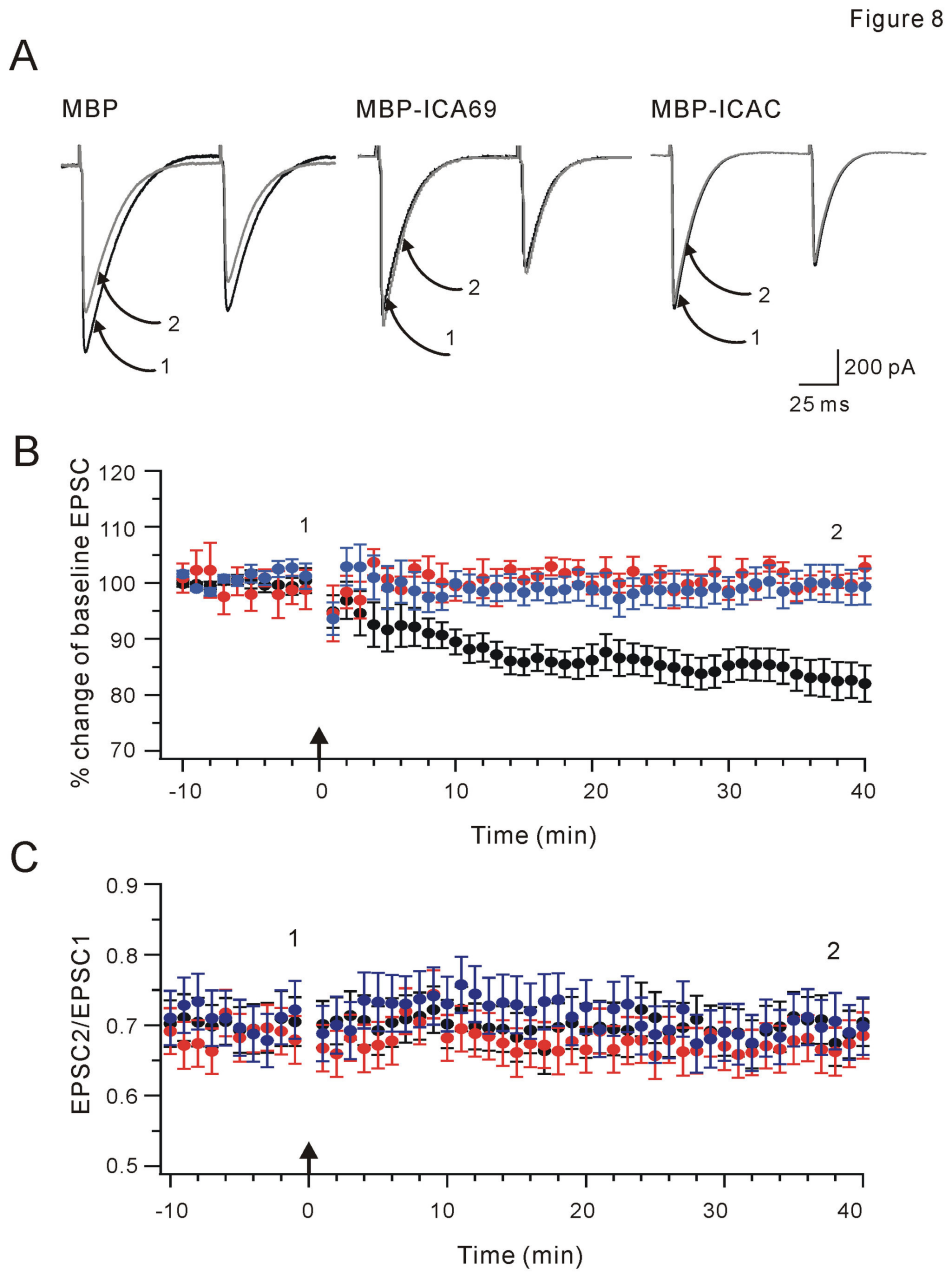
ICA69 and ICAC inhibit CF-LTD. (A) Example traces before and after CF-LTD. Note that EPSC1 was bigger than EPSC2. Currents indicated by 1 (black) or 2 (grey) were collected at times indicated in (B). (B) Comparison of three groups. Mean peak amplitudes of CF-EPSC1 are displayed versus time (Black: MBP, n = 9; Red: MBP-ICA69, n = 11; Blue: MBP-ICAC, n=9). Following a 10-min baseline recording, CF-LTD was induced at t = 0 min. Tetanic stimulation is indicated by the upward arrow. (C) Time courses of PPF of EPSCs. Black: MBP, n = 9; Red: MBP-ICA69, n = 11; Blue: MBP-ICAC, n=9.

CF-PC LTD is induced by a tetanus of 5 Hz stimuli for 30 s at CF-PC synapses [29,30]. Like PF-LTD, CF-LTD requires postsynaptic Ca^2+^ elevation, activation of group 1 metabotropic glutamate receptor, and activation of PKC [29-31]. It was of interest to investigate the effect of ICA69 in CF-LTD since it is unknown to date. As shown in Figure 8A and 8B, CF-LTD was induced by 5-Hz stimuli when MBP backbone protein (50 μg/ml) was perfused into PCs (82±3% of baseline at t = 38 min, n = 9; P <0.01). However, this LTD was significantly reduced when either MBP-ICA69 (50 μg/ml) or MBP-ICAC (50 μg/ml) was perfused into PCs. (MBP-ICA69: 100±1% of baseline at t = 38 min, n = 11; P >0.05; MBP-ICAC: 99±3% of baseline at t = 38 min, n = 9; P >0.05; Figure 8A and 8B). These results implied that, similar to PF-LTD, CF-LTD may be PICK1-dependent and subject to the inhibition by ICA69. PPF was not changed in all groups (Figure 8C). 

## Discussion

In current work, we identified that ICAC and ICAC 84-224 interacted with PICK1 and regulated the trafficking of PICK1-PKCα complex. Although both BAR and ICAC domains of ICA69 associate with PICK1, our work suggested that ICAC and ΔICAC (containing BAR domain) might be functionally distinct in the association between ICA69 and PICK1. While ΔICAC domain inclined to form clusters, the distribution of ICAC was diffuse. In view of results that PICK1 distribution is diffuse when co-expressed with ICA69 in neurons and HEK293T cells [22], we considered that ICAC might be the dominant fragment to control the pattern of PICK1 distribution. Notably, we found that the trafficking of PICK1 to membrane was inhibited by PICK1-ICA69 complex. This action might ascribe to ICAC, because the overexpression of ICAC, but not ΔICAC, interrupted PKCα-mediated PICK1 trafficking. Function of ICAC on PICK1 trafficking was further confirmed by our electrophysiological recordings, where PF-LTD and CF-LTD were blocked by the inclusion of MBP-ICA69 or MBP-ICAC in PCs during slice recordings.

PICK1 at synapses is essential for the induction of synaptic plasticity in cerebellum and hippocampus [8,9,13]. LTD is deficient in PICK1 knockout mice [9], but rescued by the overexpression of PICK1 [32]. In addition, activation of PKCα is critical for cerebellar LTD [14,33]. PICK1 interacts with PKCα [2] and activated PKCα transports PICK1 to membrane [26]. It suggests that the trafficking of PICK1-PKCα complex to membrane brings PICK1 close to GluR2 PDZ ligand and PKCα close to Ser880 to facilitate GluR2 phosphorylation. This is critical for the internalization of GluR2 and LTD induction [11]. We found that PICK1 did not accompany PKCα to membrane but was retained in cytosol when ICA69 or ICAC was co-expressed (Figure 5). This may explain our results that both PF-LTD and CF-LTD were blocked by MBP-ICA69 or MBP-ICAC included in PCs (Figure 7 and 8). PICK1 is also found to induce clustering of GluR2 and increase the number of AMPARs at synapses [3,4,34,35]. Overexpression of ICA69 significantly disrupts PICK1-mediated GluR2 clustering and decreases synaptic targeting of GluR2 [22]. It will be intriguing to examine whether ICAC also brings about similar interruption to GluR2 targeting. 

Cao et al. [22] showed that ICA69 and PICK1 are both abundantly expressed in cell bodies and dendrites of PCs, suggesting that ICA69 may play roles in PICK1-mediated synaptic scaling and plasticity. It is speculated that ICA69 may exist in only dendritic shafts but not spines [22]. Our data showed that LTD induction was inhibited when the expressions of ICA69 and ICAC were increased in PCs (Figures 7 and 8), suggesting that the balance of heteromeric complex of ICA69 and PICK1 is important for PICK1 trafficking and its-mediated AMPAR targeting. It will be interesting to investigate whether synaptic efficacy or plasticity is altered when the expression of ICA69 is altered in a transgenic animal model, for example, conditioned ICA69 knockout mice, or in a certain disease.

PICK1 interacts with AMPAR, mGluR7, GLT-1b, dopamine transporter, acid-sensing ion channel, GRIP, PKCα, β-arrestin, and Arp2/3 [18-21]. Besides, ICA69 is the major binding partner of PICK1 and co-expresses with PICK1 in many tissues, including the brain, pancreas, and testis [22]. ICA69 and PICK1 colocalize well in subcellular regions of neurons [22], suggesting that BAR domain of ICA69 may provide the ability to bind to PICK1 [22]. Strikingly, our Co-IP and FRET data indicated that ICAC was able to bind to PICK1 (Figures 2 and 3). Indeed, ICAC was highly conserved across species (Figure 1), suggesting it is critical to the normal function of ICA69. Specifically, we found that ICAC84-224 was sufficient to bind to PICK1 and retained it in the cytosol in response to TPA stimulation (Figure 6). These results may push forward in search of the precise regulatory machinery of PICK1-ICA69 complex.

An interesting finding was that the distribution of ICAC was diffuse (Figure 1). Moreover, ICAC caused a diffuse distribution of PICK1 when they were co-expressed (Figure 2). Since PICK1 formed clusters when it was co-expressed with ΔICAC (Figure S3), these results implied that ICAC and BAR domains may function distinctly in association with PICK1. This assumption was supported by the evidence from single expression or co-expression of PICK1, ICAC, and ΔICAC (Figure 1 and 2). Experiments of single expression of ICA69 truncations in 293T cells showed that there appeared to be more clusters accompanied with deletion of the longer C-terminal fragment of ICA69 (Figure 1F). Single transfection of ΔICAC displayed the most clusters (Figure 1F, first row). In contrast, single transfection of either ICAC or ICAC84-224 shows no cluster (Figure 1F, second row). It should be noted that a small percentage of cells with single ICA69 transfection showed clusters (data not shown), suggesting that ICAC and BAR affect the distribution of ICA69 to different degrees. In case of co-transfection, Cao et al. [22] found that the distribution of PICK1 is diffuse when co-expressed with ICA69, and this was confirmed in our work (Figure 2A). However, PICK1 formed more clusters when ΔICAC was co-expressed with PICK1 (Figure 6C). All these suggested that BAR and ICAC are able, but not necessary, to form the complex with PICK1. Particularly, ICAC controls the distribution pattern of PICK1-ICA69 complex when BAR and ICAC are both present, such as ICA69. On the other hand, they play distinct roles in PICK1 trafficking, as shown by our results that ICAC could retain PICK1 in cytosol (Figure 5C), but ΔICAC could not (Figure 6C). How ICAC causes the diffuse distribution of PICK1 remains to be solved.

## Materials and Methods

### Plasmid construction

All animal experiments were approved by the Animal Experimentation Ethics Committee of Zhejiang University and specifically designed to minimize the number of animals sacrificed. Animals were maintained at the Experimental Animal Center of Zhejiang University and kept under temperature-controlled conditions on a 12:12 h light/dark cycle with food and water *ad libitum*. Rat GFP-PICK1, GFP-ICA69, and GFP-ICAC were gifts from Jun Xia (Hong Kong University of Science of Technology, Hong Kong, China). PICK1 was subcloned into pCFP for FRET assay, or pRK5 for Co-IP. ICA69 truncations were amplified by PCR of cDNA encoding ICA69 and inserted into pEGFP plasmid for Co-IP and immunocytochemistry, or pYFP plasmid for FRET assay. ICA69, ICAC, and ΔICAC were also inserted into pET-MBP-3C plasmid for protein purification. PKCα was obtained from cDNA of rat brain and subcloned into mCherry-pRK5 or pRK5 plasmid. All constructs were verified by DNA sequencing. All plasmids were transformed into *E. coli* DH5α in order to get plenty of plasmids.

### HEK293T cell culture and transfection

HEK293T cells were cultured in MEM (GIBCO, Carlsbad, CA) and supplemented with 10% FBS (GIBCO), 1 mM sodium pyruvate, 100 U/ml penicillin, and 10 μg/ml streptomycin in a incubator (95%O_2_/5% CO_2_, 37°C). At 30-50% confluency, cells were transfected in OPTI-MEM (GIBCO) with plasmids using Lipofectamine 2000 (Invitrogen, Carlsbad, CA) according to manufacturer’s instructions. The transfected cells were used for further experiments after 36 hrs. 

### Immunocytochemistry

Immunofluorescent staining was performed as described previously [36]. In brief, 293T cells were fixed 36-48 hrs after transfection by 4% paraformaldehyde and 4% sucrose in PBS for 15 min at room temperature. Cells were then permeabilized by 0.2% Triton X-100 in PBS for 15 min. After blocking with 10% Bovine Serum Albumin in PBS for 1 hr, transfected 293T cells were incubated with affinity-purified mouse anti-myc (1:500) overnight and then 1 hr incubation with mouse Cy3-conjugated goat anti-mouse secondary antibody (1:800). After washing with PBS, cells were immediately fixed and imaged on a confocal microscope (Olympus, Tokyo, Japan). All processes were performed at room temperature. All parameters used in confocal microscopy were consistent in each experiment, including laser excitation power, detector, off-set gains, and pinhole diameter. Image J software (NIH, Bethesda, MD) was used to analyze cell images. Imaging data analysts were blind to the experimental conditions until the data were integrated.

### Co-immunoprecipitation

Co-IP was performed according to our previous work [36]. 293T cells or cortical tissue were lysised in RIPA buffer plus protease inhibitor mixture (Calbiochem, San Diego, CA). Protein concentration in the supernatant was determined using a BCA kit assay (Thermo, Rockford, IL) after centrifugation at 16,000×g at 4°C for 30 min. Decimus supernatant was used for input and others for immunoprecipitation. To detect the interaction of myc-PICK1 with GFP-ICA69 or its truncations, anti-GFP or anti-myc antibody was incubated with protein A-sepharose (GE Healthcare, Waukesha, WI) at 2-4 μg antibody/1 ml sepharose beads for 2 hrs in 50 mM Tris-HCl. Rabbit or mouse IgG (Invitrogen) was used as negative control. The beads were washed with a binding buffer and stored at 4°C. Pre-cleared solubilized preparations were incubated with anti-GFP antibody pre-coupled to protein A-sepharose beads. Proteins on the beads were extracted with a 2×SDS sample buffer. We boiled all samples for 5 min before Western blot assay.

### Western blot

Western blot was performed according to previous work [36,37]. Samples were loaded on tris-glycine gels. After electrophoresis, proteins were transferred to PVDF membranes (Millipore, Billerica, MA). The membranes were blocked with 5% nonfat milk and incubated with antibodies in TBS containing 0.1% tween-20. Concentrations of primary antibodies were anti-PICK1 (1:2000), anti-ICA69 (1:2000), anti-HA (1:2000), rabbit anti-GFP (1:2000), mouse anti-GFP (1:3000), anti-myc (1:2000), anti-MBP (1:3000), and anti-PKCα (1:5000). Horseradish proxidase-conjugated secondary antibodies were diluted at 1:5000. Blots reacted with chemiluminescent substrate ECL (Pierce, Rockford, IL) and exposed to film (Kodak, Rochester, NY). The film was digitally scanned, and signals on digital images were quantitated using Image-J 1.42q (NIH, Bethesda, MD). GAPDH was set as internal control.

### Detection of fluorescence resonance energy transfer (FRET)

The fluorescence imaging work station for FRET was described in previous work [38,39]. 2×2 binning modes and a 200-ms integration time were used. The average background signal was determined as mean fluorescence intensity from an area not expressing constructs and was subtracted from raw images before we carried out FRET calculations. Following fluorescence filters were used: CFP (I_CFP_: excitation, 436 nm; dichroic mirror, 455 nm; emission, 480 nm); YFP (I_YFP_: excitation, 500 nm; dichroic mirror, 515 nm; emission, 535 nm); FRET (I_FRET_: excitation, 436 nm; dichroic mirror, 455 nm; emission, 535 nm). To calculate the FRET efficiency, a normalized FRET signal (*N*_FRET_) was measured using Eq. 1 described previously [40]:

*N*_FRET_ = (I_FRET_ – a × I_FRET_ – b × I_FRET_) / sqrt (I_YFP_ × I_CFP_) …… (Eq. 1)

where a and b represented the bleed-through values for YFP and CFP, respectively.

### Time-lapse imaging

Time-lapse imaging experiments were performed as described previously [26]. The protein translocation was observed 36 hrs after transfection using time-lapse confocal microscopy (Olympus). Cells were perfused with an extracellular solution (in mM: 5 HEPES, 10 glucose, 5.4 KCl, 135 NaCl, 1.8 CaCl_2_, 1 MgCl_2_, pH 7.3). The temperature of perfusion solution was maintained at 37°C by an in-line solution heater and a TC-344B temperature controller (Warner Instruments, Hamden, CT). We used lowest intensity of lasers and shortest scanning time to minimize cell bleaching. Pinhole openings were set for a 4 μm depth of optical slice. Time-lapse images were collected at a 640×640-pixel resolution (12-bit). The interval between each scanning was set to 3 min to minimize cell bleach. Following fluorescence filters were used: mCherry (excitation: 543 nm; emission: 572 nm; bandpass filter >560 nm), GFP (excitation: 488 nm; emission: 510 nm; bandpass filter: 500-530 nm), and CFP (excitation: 405 nm; emission: 461 nm; bandpass filter: 425-475 nm). Fluorescence crosstalk was not detected in our experiments (data not shown).

Membrane fluorescence ratio was measured using MetaMorph software (Olympus), defined as the value of plasma membrane fluorescence intensity (F_m_)/cytosolic fluorescence intensity (F_cyt_) [26]. Region of interest (ROI) was circle for cytoplasm or rectangle for plasma membrane (each ~10 μm^2^). The analyzer was blind to experimental conditions and allowed to select ROIs based images at t = 0 min. For each time point, F_m_ was averaged from 2 ROIs and F_cyt_ was averaged from 3-4 ROIs. Occasionally, some cells endured slight or obvious movement. To exclude the interference of cell movement, ROI was manually moved to fit in with the middle of membrane meanwhile its shape and size were unchanged.

### Antibodies

We purchased mouse monoclonal PICK1 antibody from NeuroMab (Davis, CA). Mouse anti-HA and anti-GFP antibodies, rabbit anti-GFP and anti-PKCα antibodies were from Sigma (St. Louis, MO). Mouse anti-MBP antibody was from Shanghai Likun (Shanghai, China). Mouse anti-myc antibody was from CalBiochem. Mouse Cy3-conjugated goat anti-mouse and horseradish proxidase-conjugated secondary antibodies were from GE Healthcare. Anti-GAPDH antibody was from Abcam (Cambridge, UK). Rabbit polyclonal ICA69 and PICK1 antibodies were gifts from Jun Xia (Hong Kong). Anti-ICA69 antibody was generated against C-terminal residues 468-480 of rat ICA69.

### Purification of MBP fusion protein

The preparation of MBP fusion proteins was performed as described previously [8]. MBP, MBP-ICA69, MBP-ICAC, and MBP-ΔICAC were transformed into *E. coli* BL21 (DE3), respectively. Bacteria were grown in LB medium at 37°C until the culture reached an A_600_ of 0.5. 500 μM IPTG was added to the culture for 4 hrs and cells were harvested. Pellets were lysed in STE buffer (10 mM Tris-HCl, 150 mM NaCl, 1 mM EDTA, pH 8.0) containing 1 mg/ml lysozyme and incubated with rotation at 4°C for 1 hr. Lysates were liberated by sonication and solubilized by 2% Triton X-100 in STE buffer. Amylose resin (New England Biolabs, Hitchin, UK) was added to cleared lysates. After washing, MBP fusion proteins were eluted by STE buffer plus 10 mM maltose. Purified MBP fusion proteins were then dialyzed against internal solution. Fusion protein concentration was determined using a BCA kit assay (Thermo). SDS gel electrophoresis (SDS-PAGE) was performed on the purified MBP, MBP-ICAC, MBP-ICA69, and MBP-ΔICAC. By using Coomassie Brilliant Blue staining and Western blotting, MBP, MBP-ICAC, MBP-ICA69, and MBP-ΔICAC proteins were detected (Figure S5).

### Electrophysiology

The electrophysiological experiments were modified from previous work [28,41]. Parasagittal slices of the cerebellar vermis (250 μM) were prepared from P17-23 mice using a vibrating tissue slicer (Leica VT1000S, Germany) and ice-cold standard artificial cerebrospinal fluid (ACSF) containing (in mM): 124 NaCl, 5 KCl, 1.25 NaH_2_PO_4_, 2 MgSO_4_, 2 CaCl_2_, 26 NaHCO_3_, 10 D-glucose, bubbled with 95 O_2_ and 5% CO_2_. After a recovery period of 30 min at 37°C, slices were placed in a submerged chamber that was perfused at 2 ml/min with ACSF supplemented with 10 μM GABAzine to block GABA_A_ receptors. Recording electrodes were filled with a solution containing (in mM): 135 Cs methansulfonate, 10 CsCl, 10 HEPES, 4 Na_2_ATP, 0.4 Na_3_GTP, 0.3 EGTA (pH 7.2). Resistances of recording pipettes were typically 1.5-3 MΩ. Uncompensated series resistances were less than 5 MΩ.

 Purkinje cells were visualized using an upright Olympus BX51 microscope (Olympus) with a 40× water immersion objective and equipped with infrared-differential interference contrast enhancement. We selected Purkinje cells in lobule V for recording throughout experiments to avoid regional cholesterol difference. Whole-cell recordings were obtained with an Axopatch 200B amplifier (Molecular Devices, Foster City, CA). Currents were filtered at 1 kHz and digitized at 10 kHz. For PF stimulation, standard patch pipettes were filled with ACSF and placed in the middle third of the molecular layer [27,28]. For CF stimulation, bipolar electrodes fashioned from theta pipette glass were positioned in the granule cell layer close to the vicinity of recorded neurons, and stimulus intensity and electrode position were adjusted so that all-or-none CF responses were elicited [30,42]. When burst stimulation was employed, the interpulse interval was set as 10 ms. Paired-pulse facilitation protocol was employed to measure peak amplitudes of two consecutive EPSCs separated by a inter-stimulus interval of 80 ms. The average of three trials was used to calculate the percentage facilitation (100×(A2–A1)/A1), where A1 and A2 were the average peak amplitude of first and second EPSCs, respectively. All drugs for electrophysiology experiments were purchased from Tocris (Bristol, UK) and Sigma (St. Louis, MO). Cells were excluded from the study if series resistance or input resistance varied by more than 15% over course of an experiment.

### Data analysis

Statistics was performed using Excel 2003 (Microsoft, Chicago, IL), Clampfit 10 (Molecular Devices) and Igor Pro 6.0 (Wavemetrics, Lake Oswego, OR). Statistical differences were determined using either Student’s *t*-test for two-group comparisons or one-way ANOVA test for multiple comparisons. Statistical significance was accepted when P <0.05. Data in the text and figures are presented as mean±SEM. n represents the number of cells or experiments. 

## Supporting Information

Figure S1 **Efficiency of pull-down with GFP antibody.** (A) Myc-PICK1 and GFP-tagged ICA69 or its truncations were co-expressed in 293T cells. Constructs transfected are listed on above as “+” or “-”. Lysates were immunoprecipitated with rabbit anti-GFP antibody. Proteins from IPs were eluted and detected by Western blot using mouse anti-GFP antibody (upper panel). As controls, IgG was detected in same lysate using anti-rabbit second antibody (lower panel). (B) Ratios of GFP blot/IgG blot for all precipitated proteins were calculated and normalized to ICA69 group. Percentages were 104±9% (ICAC), 98±10% (ICAC84-224), 96±8% (ICAC1-83), and 93±8% (GFP). Results were derived from 4 independent cultures.
(TIF)

Figure S2 **TPA fails to induce translocation of either ICA69 or PICK1 when they are co-expressed in 293T cells.** GFP-ICA69 and mCherry-PKCα were co-expressed in 293T cells. As shown in 12- and 24-min images, both ICA69 and PICK1 were retained in cytosol in response to 2 μM TPA. Scale bar: 10 µm.
(TIF)

Figure S3 **ΔICAC and PICK1 colocalize when co-expressed in 293T cells.** GFP-ΔICAC and myc-PICK1 were co-transfected into 293T cells. Images derived from a representative cell show that ΔICAC and PICK1 colocalized well in 293T cells. Scale bar: 10 µm.
(TIF)

Figure S4 **ICAC1-83 does not affect TPA-induced translocation of PICK1.** (A) GFP-ICAC1-83, mCherry-PKCα, and CFP-PICK1 were co-expressed in 293T cells. Note that ICAC1-83 was diffuse, different from PICK1 and PKCα. After TPA (2 μM) treatment, PICK1 and PKCα were translocated to membrane while ICAC1-83 was still diffuse. For images at 0 and 24 min, higher magnifications of membrane (enclosed in small white boxes) showed the translocation of PKCα and PICK1. Scale bar: 10 µm. (B) At 0 min, F_m_/F_cyt_ values of GFP-ICAC1-83, mCherry-PKCα, and CFP-PICK1 were 1.04±0.07, 1.00±0.04, and 1.03±0.04, respectively (n = 82). At 24 min, F_m_/F_cyt_ values of GFP-ICAC1-83, mCherry-PKCα, and CFP-PICK1 were 0.90±0.05, 1.84±0.08, and 1.60±0.06, respectively (n = 82). **P <0.01.
(TIF)

Figure S5 **Preparation of MBP, MBP-ICAC, MBP-ICA69, and MBP-ΔICAC.** (A) Coomassie-stained SDS/PAGE gel reveals the enrichment of MBP (lane 2), MBP-ICAC (lane 3), and MBP-ICA69 (lane 4), MBP-ΔICAC (lane 5). Molecule weights of MBP, MBP-ICAC, MBP-ICA69, and MBP-ΔICAC were 45, 85, 115, and 74 kD, respectively. (B) Purified MBP, MBP-ICAC, MBP-ICA69, and MBP-ΔICAC proteins were detected by Western blots using mouse antibody against MBP. (C) Western blots of purified MBP, MBP-ICAC, MBP-ICA69, and MBP-ΔICAC using rabbit anti-ICA69 antibody. Note that MBP-ΔICAC was not blotted by ICA69 antibody because the latter was generated against C-terminal residues of ICA69.
(TIF)

Figure S6 **ΔICA69 does not affect PF-LTD.** (A) Example traces before (baseline) and after PF-LTD (t = 38 min). (B) Mean peak amplitudes of PF-evoked EPSC1 are displayed versus time (n = 11). Tetanic stimulation is indicated by the upward arrow. (C) Time courses of PPF of EPSCs.
(TIF)

Movies S1 **This movie shows time-lapse confocal images of the translocations of GFP-PICK1 and mCherry-PKCα when they were co-expressed in 293T cells.** This movie lasts for 3 s (MOV, 56 KB). Elapsed time points during imaging are labeled at bottom right. Selected frames from this movie are shown in Figure 4E. Scale bar: 10 μm.
(MOV)

Movies S2 **Time-lapse images show that GFP-ICA69 abolishes the CFP-PICK1 trafficking to plasma membrane following mCherry-PKCα in 293T cells.** This movie lasts for 3 s (MOV, 66 KB). Elapsed time points during imaging are labeled at bottom left. Selected frames from this movie are shown in Figure 5A. Scale bar: 10 μm.
(MOV)

Movies S3 **Time-lapse images show that GFP-ICAC84-224 abolishes the CFP-PICK1 trafficking to plasma membrane following mCherry-PKCα in 293T cells.** This movie lasts for 3 s (MOV, 231 KB). Elapsed time points during imaging are labeled at bottom right. Selected frames from this movie are shown in Figure 6A. Scale bar: 10 μm.
(MOV)
