# Supplementary figures and images for: C-Terminal Domain of ICA69 Interacts with PICK1 and Acts on Trafficking of PICK1-PKCα Complex and Cerebellar Plasticity
Source: PLoS One. 2013 Dec 16;8(12):e83862. doi: 10.1371/journal.pone.0083862 (PMC3865253; doi:10.1371/journal.pone.0083862)

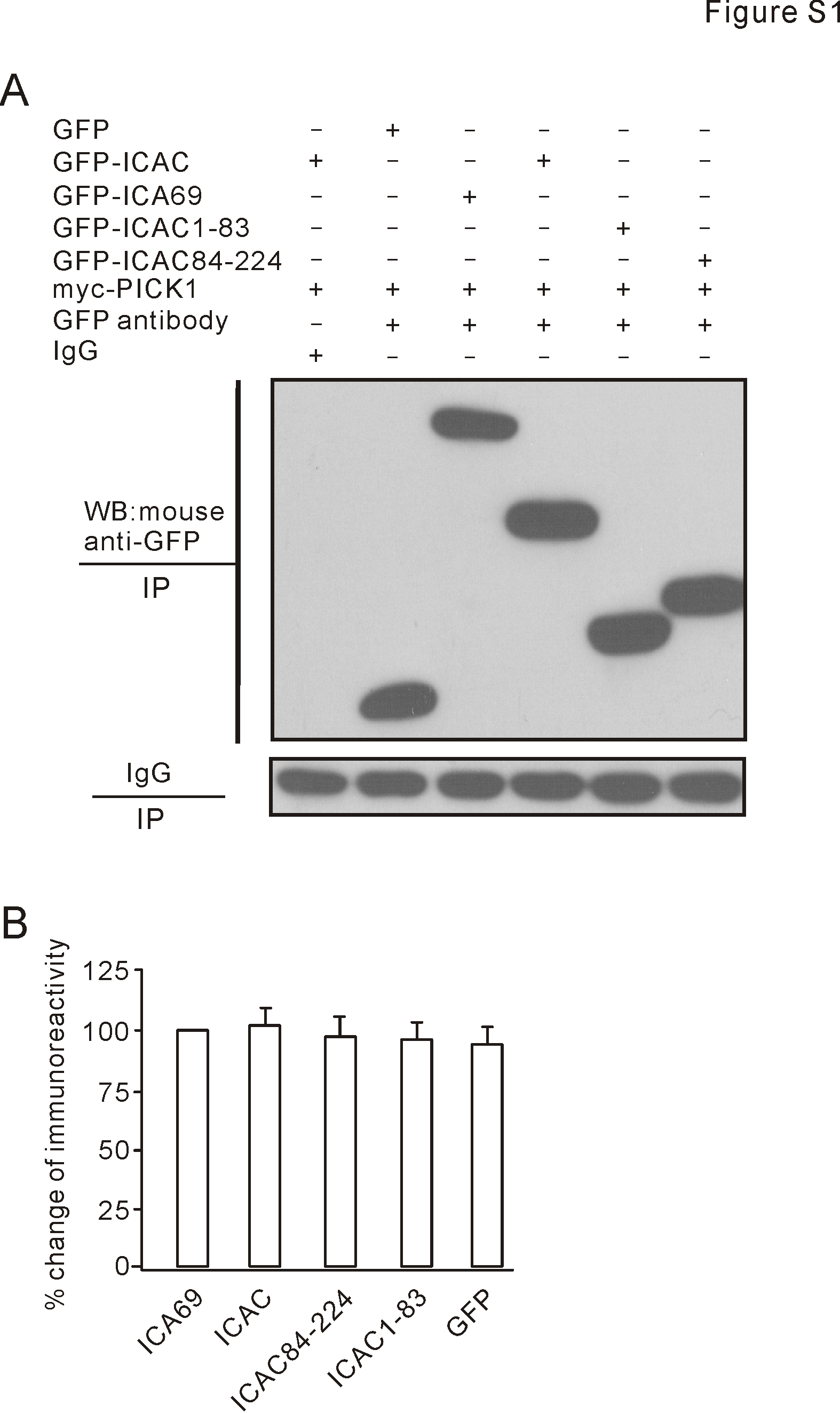

Supplement: Figure S1 — Efficiency of pull-down with GFP antibody. (A) Myc-PICK1 and GFP-tagged ICA69 or its truncations were co-expressed in 293T cells. Constructs transfected are listed on above as “+” or “-”. Lysates were immunoprecipitated with rabbit anti-GFP antibody. Proteins from IPs were eluted and detected by Western blot using mouse anti-GFP antibody (upper panel). As controls, IgG was detected in same lysate using anti-rabbit second antibody (lower panel). (B) Ratios of GFP blot/IgG blot for all precipitated proteins were calculated and normalized to ICA69 group. Percentages were 104±9% (ICAC), 98±10% (ICAC84-224), 96±8% (ICAC1-83), and 93±8% (GFP). Results were derived from 4 independent cultures. (TIF) [file pone.0083862.s001.tif]

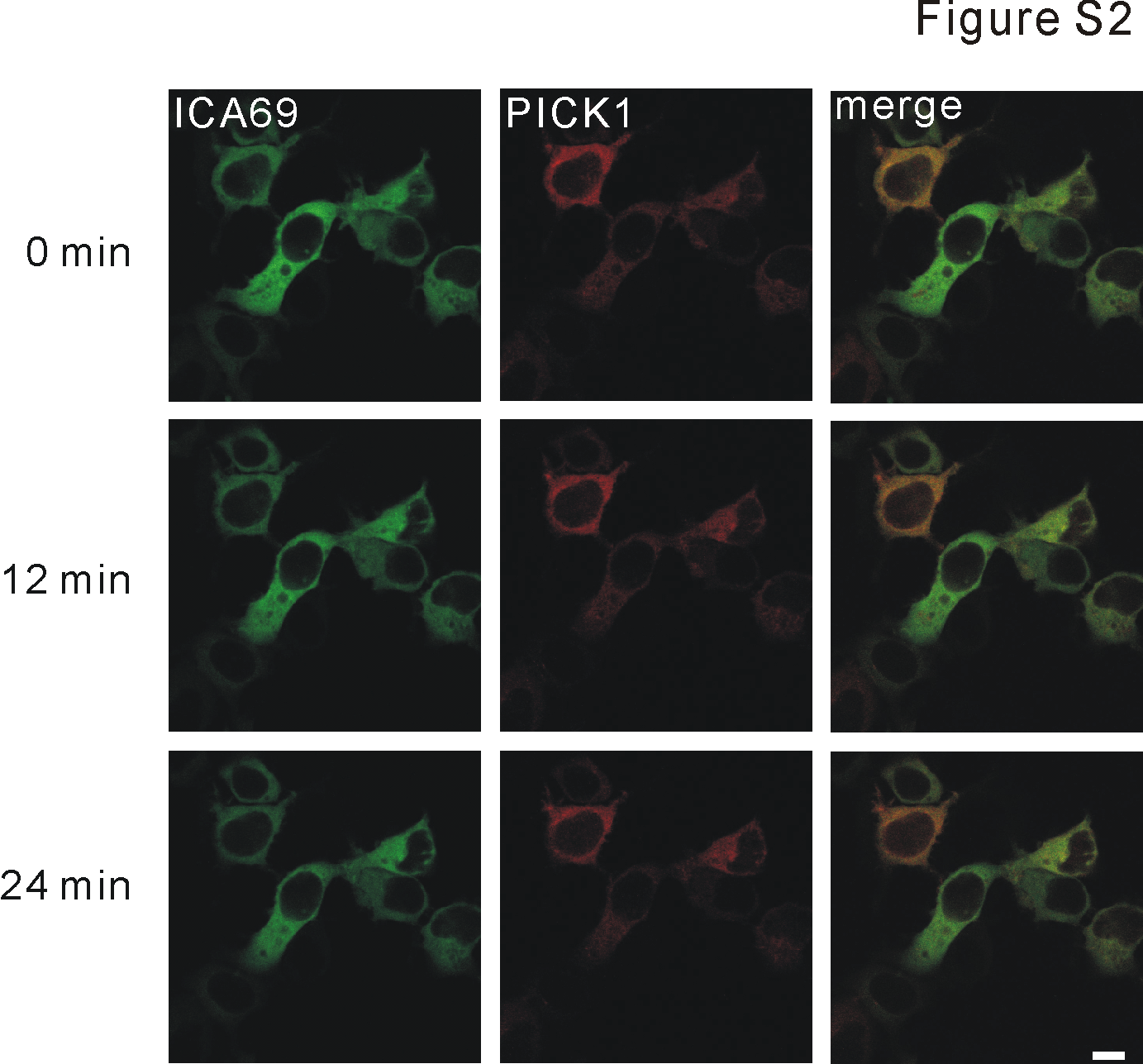

Supplement: Figure S2 — TPA fails to induce translocation of either ICA69 or PICK1 when they are co-expressed in 293T cells. GFP-ICA69 and mCherry-PKCα were co-expressed in 293T cells. As shown in 12- and 24-min images, both ICA69 and PICK1 were retained in cytosol in response to 2 μM TPA. Scale bar: 10 µm. (TIF) [file pone.0083862.s002.tif]

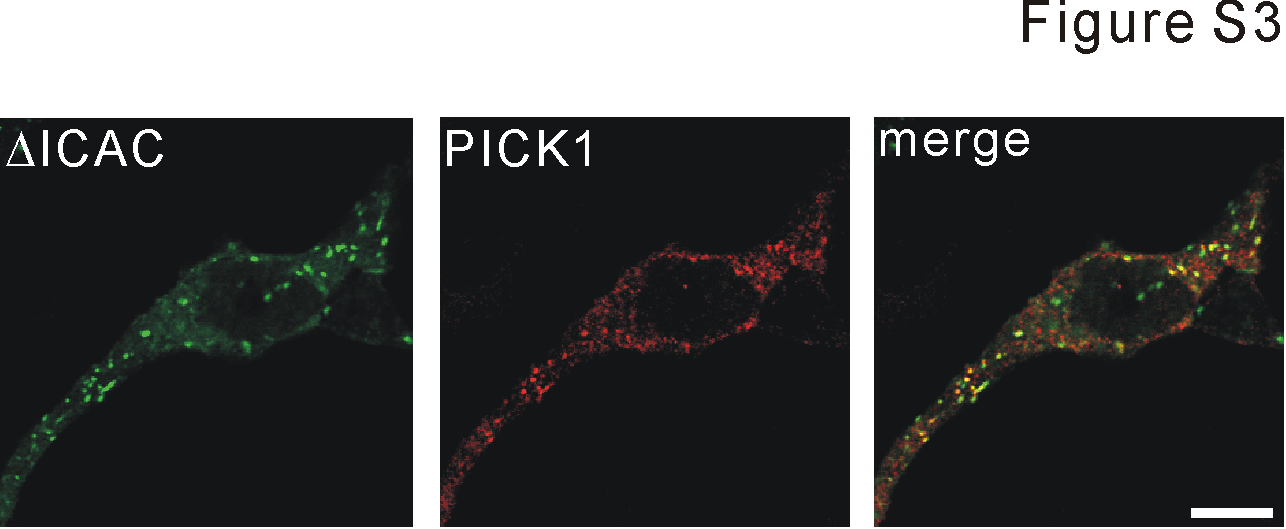

Supplement: Figure S3 — ΔICAC and PICK1 colocalize when co-expressed in 293T cells. GFP-ΔICAC and myc-PICK1 were co-transfected into 293T cells. Images derived from a representative cell show that ΔICAC and PICK1 colocalized well in 293T cells. Scale bar: 10 µm. (TIF) [file pone.0083862.s003.tif]

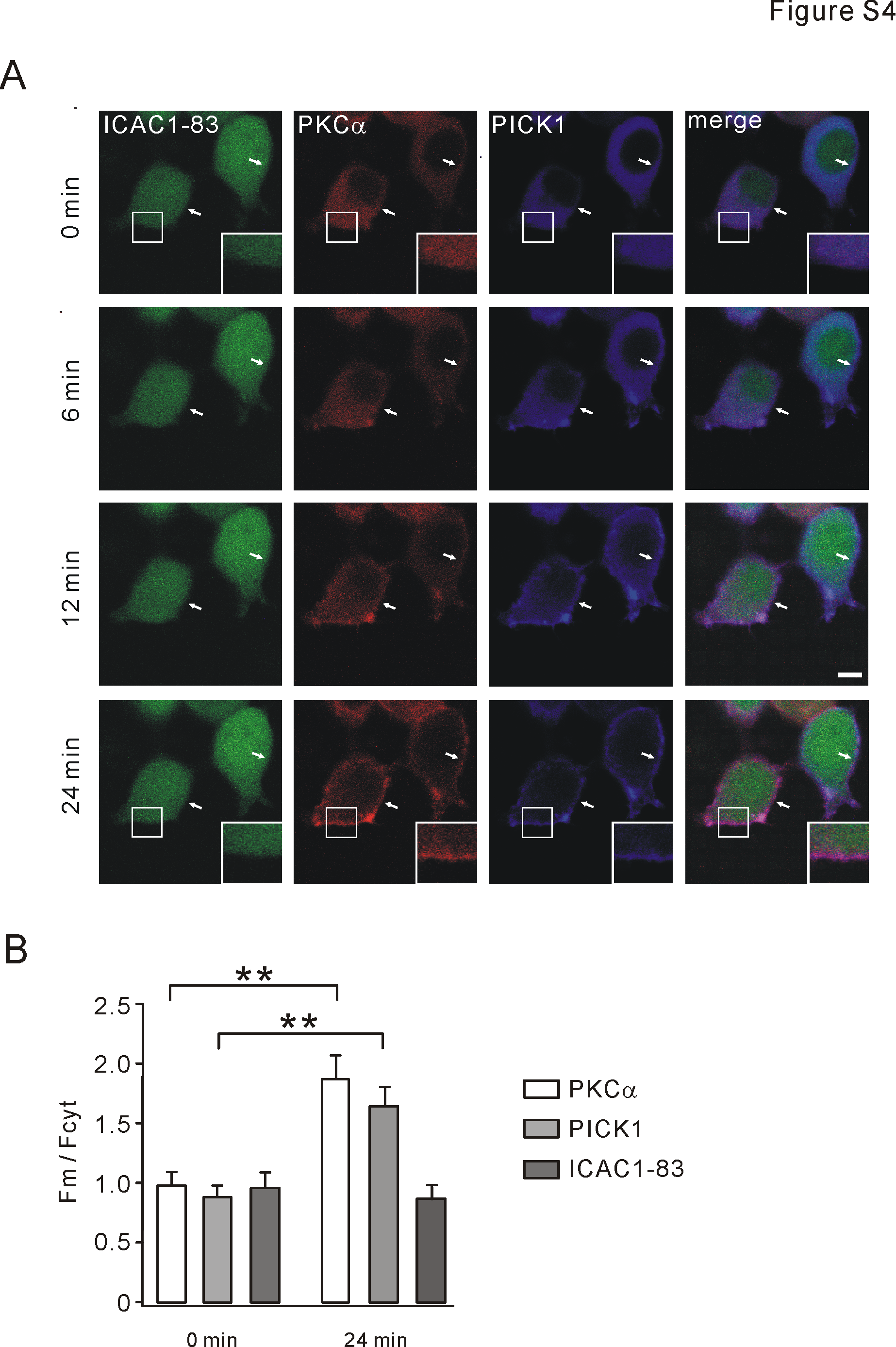

Supplement: Figure S4 — ICAC1-83 does not affect TPA-induced translocation of PICK1. (A) GFP-ICAC1-83, mCherry-PKCα, and CFP-PICK1 were co-expressed in 293T cells. Note that ICAC1-83 was diffuse, different from PICK1 and PKCα. After TPA (2 μM) treatment, PICK1 and PKCα were translocated to membrane while ICAC1-83 was still diffuse. For images at 0 and 24 min, higher magnifications of membrane (enclosed in small white boxes) showed the translocation of PKCα and PICK1. Scale bar: 10 µm. (B) At 0 min, Fm/Fcyt values of GFP-ICAC1-83, mCherry-PKCα, and CFP-PICK1 were 1.04±0.07, 1.00±0.04, and 1.03±0.04, respectively (n = 82). At 24 min, Fm/Fcyt values of GFP-ICAC1-83, mCherry-PKCα, and CFP-PICK1 were 0.90±0.05, 1.84±0.08, and 1.60±0.06, respectively (n = 82). **P <0.01. (TIF) [file pone.0083862.s004.tif]

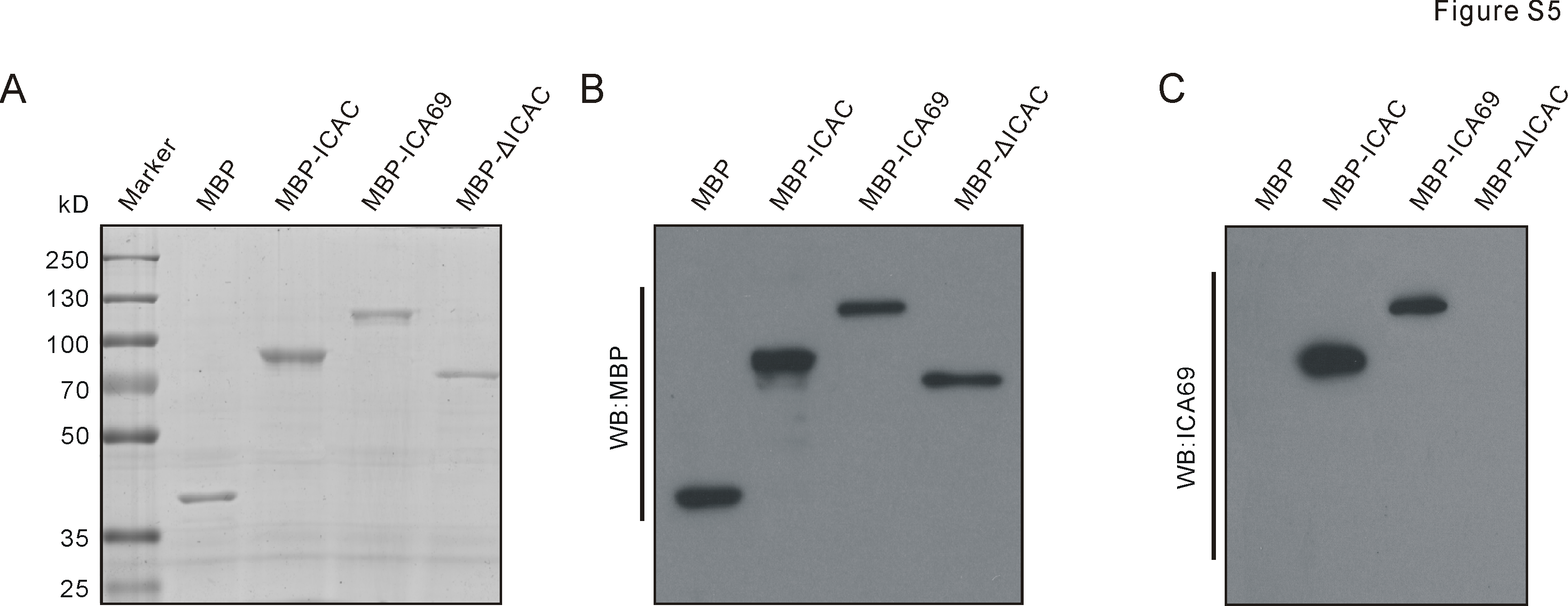

Supplement: Figure S5 — Preparation of MBP, MBP-ICAC, MBP-ICA69, and MBP-ΔICAC. (A) Coomassie-stained SDS/PAGE gel reveals the enrichment of MBP (lane 2), MBP-ICAC (lane 3), and MBP-ICA69 (lane 4), MBP-ΔICAC (lane 5). Molecule weights of MBP, MBP-ICAC, MBP-ICA69, and MBP-ΔICAC were 45, 85, 115, and 74 kD, respectively. (B) Purified MBP, MBP-ICAC, MBP-ICA69, and MBP-ΔICAC proteins were detected by Western blots using mouse antibody against MBP. (C) Western blots of purified MBP, MBP-ICAC, MBP-ICA69, and MBP-ΔICAC using rabbit anti-ICA69 antibody. Note that MBP-ΔICAC was not blotted by ICA69 antibody because the latter was generated against C-terminal residues of ICA69. (TIF) [file pone.0083862.s005.tif]

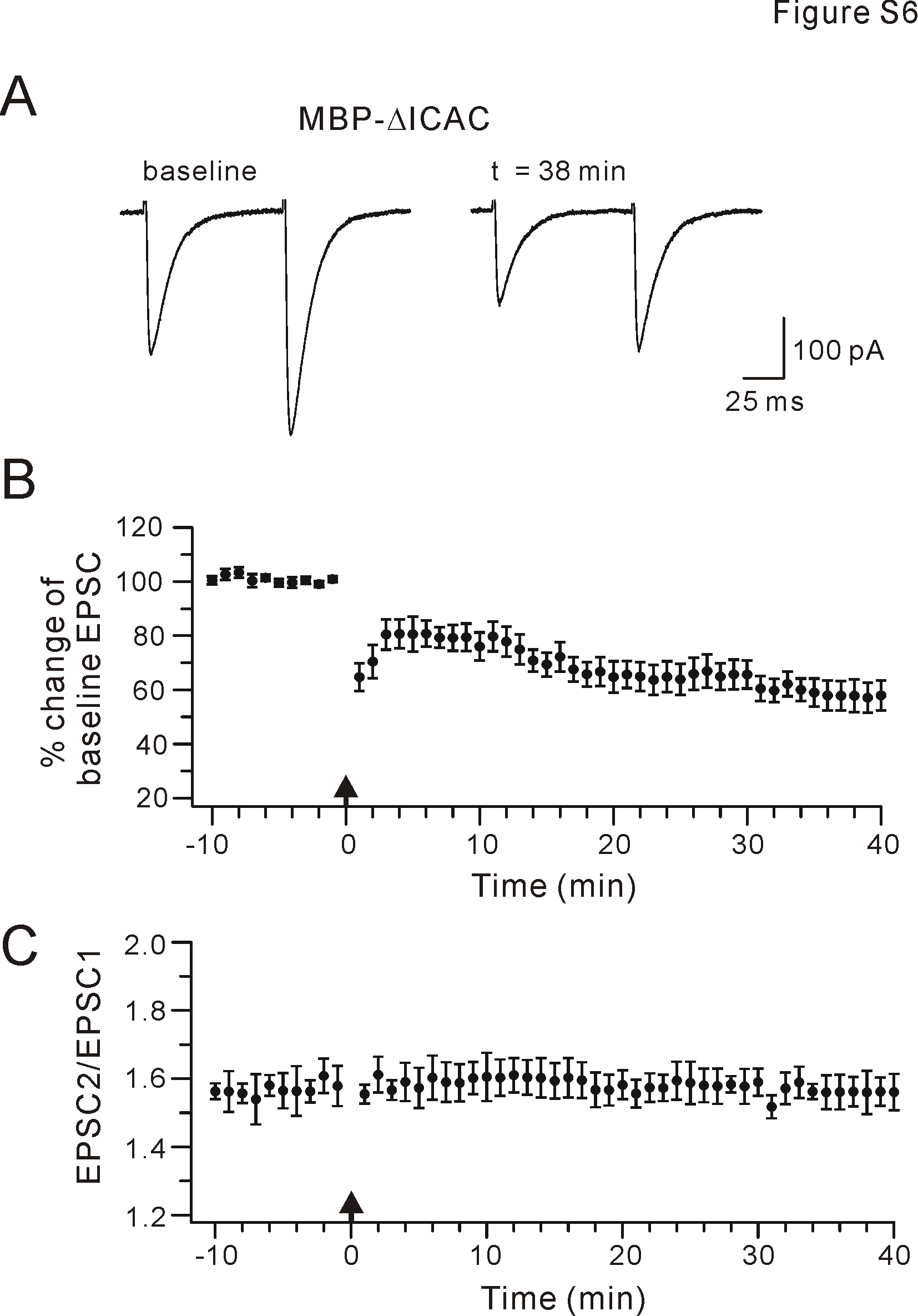

Supplement: Figure S6 — ΔICA69 does not affect PF-LTD. (A) Example traces before (baseline) and after PF-LTD (t = 38 min). (B) Mean peak amplitudes of PF-evoked EPSC1 are displayed versus time (n = 11). Tetanic stimulation is indicated by the upward arrow. (C) Time courses of PPF of EPSCs. (TIF) [file pone.0083862.s006.tif]
